# The Burden of Hepatitis A Outbreaks in the United States: Health Outcomes, Economic Costs, and Management Strategies

**DOI:** 10.1093/infdis/jiae087

**Published:** 2024-02-28

**Authors:** Emily K Horn, Oscar Herrera-Restrepo, Anna M Acosta, Alyssa Simon, Bianca Jackson, Eleanor Lucas

**Affiliations:** GSK, Philadelphia, Pennsylvania, USA; GSK, Philadelphia, Pennsylvania, USA; GSK, Philadelphia, Pennsylvania, USA; OPEN Health Group, Hingham, Massachusetts, USA; OPEN Health Group, Hingham, Massachusetts, USA; OPEN Health Group, Hingham, Massachusetts, USA

**Keywords:** HepA outbreaks, HepA vaccination, health outcomes, economic burden, outbreak management

## Abstract

**Background:**

Hepatitis A (HepA) vaccines are recommended for US adults at risk of HepA. Ongoing United States (US) HepA outbreaks since 2016 have primarily spread person-to-person, especially among at-risk groups. We investigated the health outcomes, economic burden, and outbreak management considerations associated with HepA outbreaks from 2016 onwards.

**Methods:**

A systematic literature review was conducted to assess HepA outbreak-associated health outcomes, health care resource utilization (HCRU), and economic burden. A targeted literature review evaluated HepA outbreak management considerations.

**Results:**

Across 33 studies reporting on HepA outbreak-associated health outcomes/HCRU, frequently reported HepA-related morbidities included acute liver failure/injury (n = 6 studies of 33 studies) and liver transplantation (n = 5 of 33); reported case fatality rates ranged from 0% to 10.8%. Hospitalization rates reported in studies investigating person-to-person outbreaks ranged from 41.6% to 84.8%. Ten studies reported on outbreak-associated economic burden, with a national study reporting an average cost of over $16 000 per hospitalization. Thirty-four studies reported on outbreak management; challenges included difficulty reaching at-risk groups and vaccination distrust. Successes included targeted interventions and increasing public awareness.

**Conclusions:**

This review indicates a considerable clinical and economic burden of ongoing US HepA outbreaks. Targeted prevention strategies and increased public awareness and vaccination coverage are needed to reduce HepA burden and prevent future outbreaks.

## Teaser

Ongoing hepatitis A outbreaks in the United States are associated with a substantial clinical and economic burden. Prevention strategies and efforts to increase awareness and vaccination coverage are needed to reduce the burden of hepatitis A and prevent future outbreaks.

## Graphical Abstract

**Graphical Abstract:**
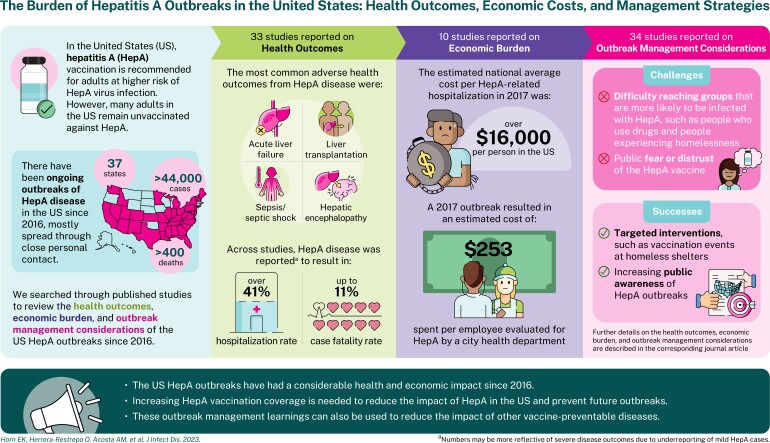

The ongoing hepatitis A (HepA) virus outbreaks across the United States (US) have spread primarily via person-to-person transmission since 2016, prior to which large outbreaks were often attributable to food contamination [1–3]. People who use drugs, people experiencing homelessness, people who are or were recently incarcerated, men who have sex with men (MSM), and recent international travelers are at highest risk of HepA infection [1–3]. While HepA infection typically results in short-term illness, among some, complications may be severe, ranging from relapsing hepatitis to acute liver failure and even death [4, 5].

Although treatment of HepA remains limited to symptom management and supportive care, infection is preventable with approved HepA vaccines, which first became available in the United States in 1995 [6, 7]. Since 2006, the Centers for Disease Control and Prevention's (CDC) Advisory Committee on Immunization Practices (ACIP) has recommended routine HepA vaccination for all children aged ≥1 year [8]. The ACIP also recommends HepA vaccination for adults at risk of HepA infection or at increased risk of severe disease from HepA infection, as well as adults who want protection against HepA without known risk factors [9]. However, HepA vaccination coverage among US adults remains low [9, 10]. In 2018, only 11.9% of adults self-reported receiving the full HepA vaccine series (≥2 doses) [11], compared with 79.6% coverage among infants by age 35 months (2019–2021) and 84.8% among adolescents aged 13–17 years (2022), as reported by their vaccination providers [12, 13].

Between 2016 and 2023, over 44 000 HepA cases, of which 61% required hospitalization, and over 400 deaths were reported across 37 states [2]. However, infections especially in younger children are typically not accompanied by symptoms, likely resulting in underreporting of cases and consequential overestimations of hospitalization and case fatality rates in the overall population [4]. Nonetheless, the health outcomes and associated health care resource utilization (HCRU) related to these outbreaks incur a considerable economic cost to health care systems [14].

In light of the recent HepA outbreaks and the well-recognized gap in adult HepA vaccination, this study aimed to understand current health outcomes, economic burden, and outbreak management considerations associated with US HepA outbreaks since 2016 to help inform decision makers on the value of HepA prevention.

## METHODS

### Study Design

A systematic literature review (SLR) was conducted in accordance with the Preferred Reporting Items for Systematic Reviews and Meta-Analyses (PRISMA) guidelines to summarize the evidence regarding the health outcomes, HCRU, and economic burden associated with the ongoing HepA outbreaks [15]. A targeted literature review (TLR) was also conducted to evaluate key outbreak management considerations related to the HepA outbreaks.

### Data Collection

A comprehensive set of search terms was combined to search across MEDLINE and Embase on 12 July 2022 (Supplementary Tables 1 and 2 for the SLR and TLR, respectively). The searches were supplemented by Google, government websites, and grey literature (guidelines, commentaries, press releases, and government agency, health agency, and nongovernmental organization reports) searches.

Eligible articles for the SLR were title-/abstract- and full-text screened by 2 independent reviewers (A.S., B.J.), followed by a third reviewer (E.L.) if consensus could not be reached. Articles included in the TLR were screened and identified by 1 independent reviewer (A.S., B.J., E.L.), with 10% of articles checked by a second reviewer (A.S., B.J., E.L.) for quality control. Assessment of publication bias was not conducted.

Studies included in the SLR reported on health outcomes of interest (morbidity, mortality, and HCRU) and/or economic burden (direct, indirect, and public health intervention costs) among adult populations aged ≥18 years living in an area with a HepA outbreak from 2016 onwards (Supplementary Table 3). While studies reporting outcomes among individuals of all ages were included, this review included data only among those aged ≥18 years. Studies reporting on 1 or more key research questions associated with HepA outbreak management (successes, challenges, and key learnings) were included in the TLR. Characteristics and outcomes of included studies were qualitatively described; no statistical analyses were performed.

## RESULTS

**Figure 1.**
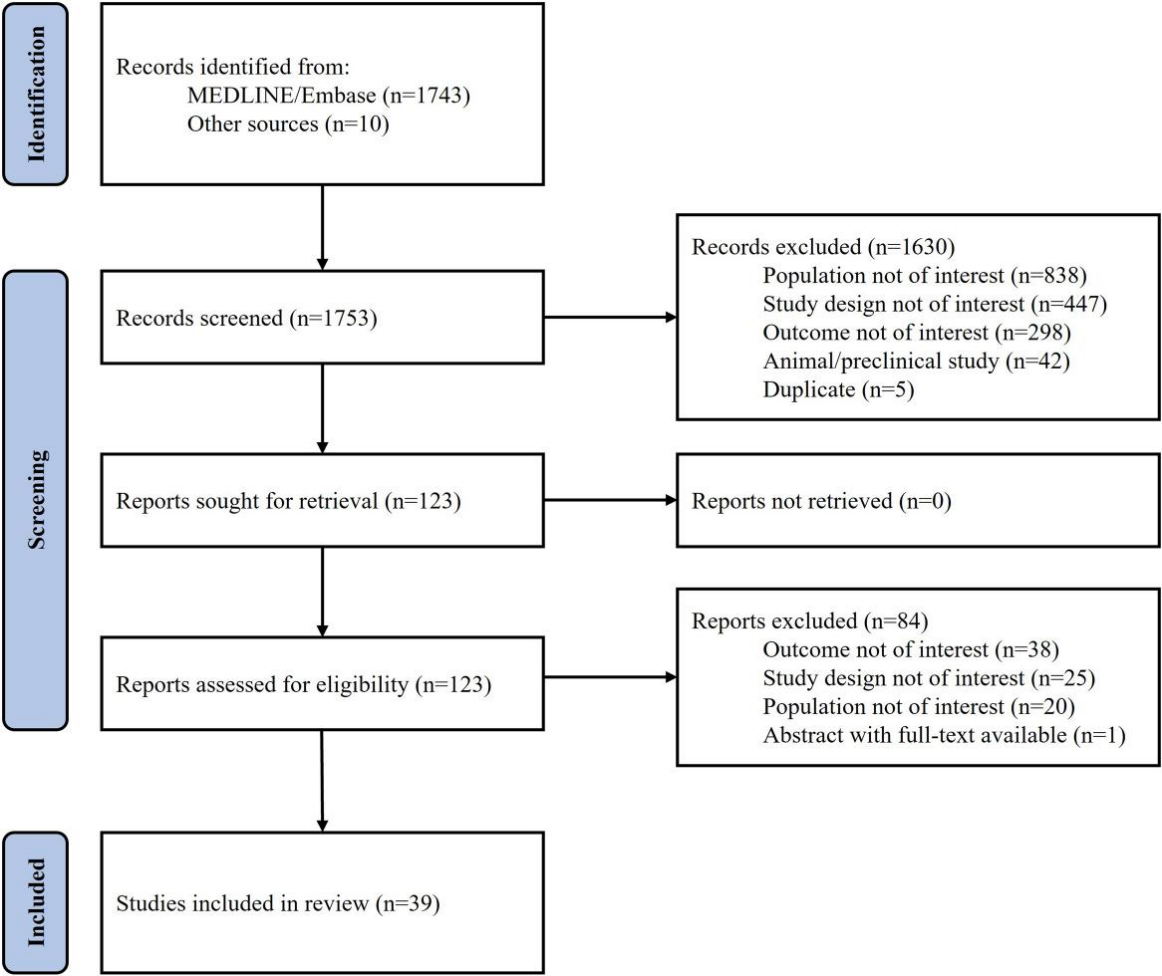
PRISMA (Preferred Reporting Items for Systematic Reviews and Meta-Analyses) flow chart.

Of the 1753 studies identified in the SLR, 1630 were excluded following title/abstract screening. Full texts of the remaining 123 studies were reviewed, and 39 were included (Figure 1). Study characteristics and patient populations of included studies are presented in Supplementary Table 4.

### Health Outcomes and Health Care Resource Utilization

The SLR identified 33 studies reporting on HepA outbreak-associated health outcomes and HCRU (Table 1). These studies reported on 1 state or area within a state (n = 18 studies), multistate (n = 7), or national (n = 8) data. Most studies reporting mode of transmission described HepA cases spread solely through person-to-person contact (n = 11), compared with solely food-borne transmission (n = 3) or both (n = 5).

**Table 1.** Health Outcomes and Health Care Resource Utilization Associated With the US HepA Outbreaks

Reference	Location, Study Population (N Cases)	Subgroup	Morbidity, n/N (%)	Case Fatality, n/N (%)	HCRU			
					Rate of Any Hospitalization, n/N (%)	LOS, Mean (SD^a^)	Rate of ICU Admission, n/N (%)	Outpatient Visits, ED Visits, 30-d Readmission Rates and Treatment Utilization, n/N (%)
Altamimi 2018 [16]	Health System within Southeast MI All HepA-positive cases that presented to Henry Ford Health System from August 2016 to December 2017 (N = 166)	Overall	Liver transplant: 1/166 (0.6)	18/166 (10.8)	122/166 (73.5)	NR	44/166 (26.5)	30-d readmission rate: 7/166 (4.2)
Brouwer 2020 [17]	MI All positive HepA cases reported to the MDSS between August 2016 and December 2018 (N = 910)	Overall	NR	30/910 (3.0)	731/910 (80.3)	NR	NR	NR
Butt 2020 [18]	Charleston, WV All positive HepA cases hospitalized at a tertiary care center between March 2017 and November 2018 (N = 482)	Overall	Sepsis: 44/482 (9.1) Encephalopathy: 17/482 (3.9)	3/482 (0.6)	482/482 (100.0)	2.5 d (4.1)	NR	NR
CDC 2021 [19]	National HepA cases reported to CDC through the NNDSS (2019: N = 18 846)	Overall	NR	2019: 225/18 846 (1.2)	NR	NR	NR	NR
CDC 2020 [20]	National HepA cases reported to the CDC through the NNDSS (2018: N = 12 474)	Overall	NR	2018: 171/12 274 (1.4)	6292/NR (58.0^b^)	NR	NR	NR
CDC 2019 [21]	National HepA cases reported to the CDC through the NNDSS (2017: N = 3366)	Overall	NR	2017: 91/3366 (2.7)	1154/1717 (67.2)	NR	NR	NR
CDC 2018 [22]	National HepA cases reported to the CDC through the NNDSS (2016: N = 2007)	Overall	NR	Death as a result of HepA: 7/1026 (0.7) Any death: 70/2007 (3.5)	456/1097 (41.6)	NR	NR	NR
CDC 2022 [2]	National State-reported HepA cases to the CDC (N = 44 660)	Overall	NR	417/44 660 (0.9)	27 280/44 660 (61.1)	NR	NR	NR
CDC 2022^c^ [23]	AZ, CA, MN, ND State-reported outbreak-associated HepA cases to the CDC linked to fresh organic strawberries (N = 19)	Overall	NR	0/19 (0)	13/19 (68.4)	NR	NR	NR
CDC 2022^c^ [24]	AZ, CA, MD, NY, NC, OR, VA, WV, WI State-reported outbreak-associated HepA cases to the CDC linked to frozen strawberries (N = 143)	Overall	NR	0/143 (0)	56/143 (39.2)	NR	NR	NR
Croker 2018 [25]	Los Angeles County, CA Patients in the outbreak unit of the hospital with HepA during July 2017 (N = 4)	Overall	NR	NR	4/4 (100.0)	Range: 0–2 d	NR	NR
Dankwa 2021 [26]	Louisville, KY HepA cases captured by surveillance conducted by the LMPHW (N = 501)	Overall	NR	NR	331/501 (66.1)	NR	NR	NR
		2017	NR	0/42 (0)	32/42 (76.2)	NR	NR	NR
		2018	NR	6/458 (1.3)	299/458 (65.3)	NR	NR	NR
		2019	NR	0/1 (0)	0/1 (0.0)	NR	NR	NR
Foster 2018 [27]	National Outbreak-associated HepA infections reported by states to the CDC through the NNDSS and/or directly to the viral hepatitis outbreak response team (2007–2011: N = 521, 2012–2017: N = 2323)	2012–2017	NR	43/2323 (2.0)	1306/2162 (60.4)	NR	NR	NR
Foster 2018 [28]	CA, KY, MI, UT Outbreak-associated HepA cases in CA, KY, MI, UT reported to the CDC in 2017 (N = 1521)	Overall	NR	41/1521 (3.0)	1073/1521 (70.5)	NR	NR	NR
		CA	NR	21/682 (3.0)	442/682 (64.8)	NR	NR	NR
		KY	NR	0/59 (0)	45/59 (76.3)	NR	NR	NR
		MI	NR	20/632 (3.0)	508/632 (80.4)	NR	NR	NR
		UT	NR	0/148 (0)	78/148 (52.7)	NR	NR	NR
Foster 2021 [29]	CA, CO, GA, MD, NY, NC, PA, VA HepA cases in CA, CO, GA, MD, NY, NC, PA, VA among MSM reported to the CDC from 1 January 2017–31 October 2018 (N = 260)	Overall	NR	0/260 (0)	124/258 (48.1)	NR	NR	NR
Haddix 2020 [30]	Los Angeles County, CA Outbreak-associated HepA cases identified by the Los Angeles County Department of Health between October 2018 and April 2019 (outbreak case definition: N = 7, surveillance case definition: N = 10)	Group with outbreak definition^b^	NR	0/7 (0)	5/7 (71.4)	NR	NR	NR
		National surveillance^d^	NR	NR	7/10 (70.0)	NR	NR	NR
Hagan 2022 [31]	AZ, AR, CA (San Diego), CO, DE, FL, GA, IL, LA, MA, MI, MN, MS, NV (Southern NV Health District), NH, NM, NY (excluding NYC), NC, OH, PA (Philadelphia), TN, UT, VT, VA, WA Outbreak-associated HepA cases reported by state and local health departments to a CDC survey from January 2016 to 24 January 2020 (N = 18 327)	Recent incarceration	NR	10/2093 (0.5)	1033/2093 (49.4)	NR	NR	NR
		Without recent incarceration	NR	199/16 234 (1.2), *P* = .003^e^	11 490/16 234 (70.8)	NR	NR	NR
Hofmeister 2020 [14]	National All HepA-related hospitalizations captured in the 2017 Healthcare Cost and Utilization Project NIS (sample size NR)	Overall	Liver transplant: NR/NR (NR); < 10 hospitalizations associated with liver transplantation	NR	NR	NR	NR	NR
Hofmeister 2021 [32]	KY, MI, WV Residents of KY, MI, or WV designated by the respective state health department as a person-to-person outbreak-associated HepA case with onset between 1 July 2016 and 10 June 2019 (N = 817) (with available information, N = 460)	Overall	Fulminant hepatitis: 20/460 (4.3); Liver transplant: 1/466 (0.2)	7/719 (1.0)	423/817 (51.8)	Mean (SE): 5.0 (0.3) Median (range): 4 (1–59) d	40/407 (9.8)	NR
		KY	Fulminant hepatitis: 9/228 (3.9); Liver transplant: 0/427 (0)	5/472 (1.1)	218/472 (46.2)	Mean (SE): 5.4 (0.4) Median (range): 4 (1–59)	20/208 (9.6)	NR
		MI	Fulminant hepatitis: 9/76 (11.8); Liver transplant: 1/92 (1.3)	2/92 (2.2)	78/92 (84.8)	Mean (SE): 4.7 (0.4) Median (range): 3.5 (1.0–22.0)	12/73 (16.4)	NR
		WV	Fulminant hepatitis: 2/156 (1.3); Liver transplant: 0/253 (0)	0/253 (0)	127/253 (50.2)	Mean (SE): 4.6 (0.4) Median (range): 4.0 (1.0–40.0)	8/126 (6.3)	NR
Hofmeister 2021 [33]	KY, MI, WV Residents of KY, MI, or WV designated by the state health department as a person-to-person outbreak-associated HepA case between 1 July 2016 and 10 June 2019 (N = 524; cases, N = 110; controls, N = 414)	Cases^f^	ALF: 74/110 (80.4) Liver transplant: 1/110 (1.1)	NR	103/108 (95.4)	1–3 d: 15/101 (14.9) 4–7: 28/101 (27.7) 8–14: 28/101 (27.7) ≥ 15: 30/101 (29.7)	81/110 (83.5)	NR
		Controls^g^	ALF: 14/414 (5.3) Liver transplant: 0/414 (0.0)	NR	248/405 (61.2)	1–3 d: 85/245 (34.7) 4–7: 110/245 (44.9) 8–14: 37/245 (15.1) ≥ 15: 13/245 (5.3)	26/414 (11.1)	NR
Hosseini 2018 [34]	San Diego County, CA Patients with HepA identified from epidemiological data provided by the San Diego Public Health Department and treated at the University of California San Diego (N = 588)	Overall	Cholestatic hepatitis: NR/154 (5.0) Clinically relapsing hepatitis: NR/154 (10.0)	20/588 (3.4)	403/588 (68.5)	NR	NR	NR
Ismail 2020 [35]	KY Adults admitted from the ED to the University of KY with acute HepA (N = 307)	Overall	Unfavorable outcome^b^: 71/307 (23.0)	NR	307/307 (100.0)	NR	71/307 (23.1)	ED visits: 307/307 (100)
Jiang 2019 [36]	San Diego County, CA All HepA cases hospitalized at the University of California San Diego Hillcrest Hospital from 1 November 2016, to 10 October 2017 (N = 106)	Overall	ALF/injury: 11/106 (10.4) Septic shock leading to death: 5/106 (4.7)	7/106 (6.6)	106/106 (100.0)	NR	NR	NR
		Patients with ALF	NR	7/11 (63.6)	NR	NR	11/11 (100)	Treatment utilization: NAC therapy: 5/11 (45.5) Mechanical ventilation: 3/11 (27.3) Renal replacement therapy: 4/11 (36.4) Vasopressor therapy: 5/11 (45.5)
		Patients with ALF on admission admitted directly to ICU	NR	NR	NR	NR	5/11 (45.5)	NR
		Patients diagnosed with ALF during hospital stay and transferred to ICU	NR	NR	NR	NR	5/11 (45.5)	NR
		Patient with ALF on admission and transferred to ICU	NR	NR	NR	NR	1/11 (9.1)	NR
		Patients without ALF	NR	NR	NR	NR	NR	Treatment utilization: NAC therapy: 18/95 (18.8) Mechanical ventilation: 3/95 (3.2) Renal replacement therapy: 2/95 (2.1) Vasopressor therapy: 3/95 (3.2)
Kaigh 2020 [37]	Philadelphia, PA All ED visits that were evaluated for HepA before and after a vaccination program from 16 July to 8 October 2019 (prevaccination program: 67 admitted, 73 diagnosed with HepA; postvaccination program: 31 admitted, 38 diagnosed with HepA)	Overall	NR	NR	NR	NR	NR	NR
		Prevaccination program	NR	NR	67/73 (91.8)	NR	NR	ED visits: 73/73 (100)
		Postvaccination program	NR	NR	31/38 (81.6)	NR	NR	ED visits: 38/38 (100)
Kang 2020 [38]	San Diego County, CA Confirmed and probable outbreak-associated cases of HepA in San Diego County from 1 November 2016 to 31 October 2018	Overall	NR	NR	119/144 (82.6)	Mean: 6 d Median (range): 3 (1–57)	NR	NR
Kreshak 2018 [39]	San Diego, CA All HepA-positive patients that presented to a tertiary care university hospital system's 2 EDs (1 urban and 1 suburban) between 1 November 2016 and 28 February 2018 (N = 133)	Overall	NR	NR	112/133 (84.2)	Median: 3 d	9/133 (6.8)^d^	ED visits: 133/133 (100) 30-d readmission rates: NR/NR (23.4)
		Surviving cohort	NR	NR	NR	Median: 3 d	NR	NR
		Deceased cohort	Sepsis as cause of death, 3/8 (37.5) ALF/injury as cause of death: 5/8 (62.5)	8/133 (6.0) Cause of death: Hemorrhagic shock: 1/133 (0.8) Sepsis: 3/133 (2.3) Non-ST-elevation myocardial infarction: 1/133 (0.8) ALF: 5/133 (3.8)	NR	Median: 18.5 d	NR	NR
Lee 2020 [40]	National Hospitalized patients identified from the 2011 to 2017 NIS with acute HepA infection stratified by survival status (N = 11 740)	Deceased cohort	Ascites: NR/114 (7.0) Sepsis: NR/114 (43) Acute kidney injury: NR/114 (57.9) Malnutrition: NR/114 (27.2) Hepatic Encephalopathy: NR/114 (3.5) ALF/injury: NR/114 (18.4)	114/11 740 (1.0)	NR (100.0)	13 d	NR	NR
		Surviving cohort	Ascites: NR/11 626 (0.8) Sepsis: NR/11 626 (7.9) Acute kidney injury: NR/11 626 (14.6) Malnutrition: NR/11 626 (6.6) Hepatic Encephalopathy: NR/11 626 (0.4) ALF/injury: NR/11 626 (1.1)	NR	NR (100.0)	7.35 d	NR	NR
Oller 2021 [41]	Lexington, KY Persons hospitalized for acute HAV at the University of KY from 4 October 2018 to 1 July 2019 (N = 31)	Overall	NR	NR	31/31 (100.0)	NR	NR	NR
		Non-Bup/NX users	NR	NR	14/14 (100.0)	12.8 (4.5)	NR	NR
		Bup/NX users	Decompensated cirrhosis: 0/17 (0) Severe hepatic encephalopathy:0/17 (0) Fulminant hepatic failure: 0/17 (0)	NR	17/17 (100.0)	23.8 (5.3)	0/17 (0)	NR
Peak 2020 [42]	San Diego County, CA Positive HepA cases in San Diego County from November 2016 to May 2018 (N = 589)	Overall	NR	20/589 (3.4)^h^	404/589 (68.6)	NR	NR	NR
Reichenbach 2021 [43]	Philadelphia, PA Positive HepA cases identified via EMR from May 2017 to December 2019 (N = 205)	Overall	Acute-on-chronic liver failure: 2/205 (1.0)^i^ ALF/injury: 2/205 (1.0) Liver transplant: 2/205 patients (1.0) with ALF evaluated for transplant but deemed not to be eligible candidates by the transplant committee due to severity of their illness including the need for multiple vasopressive agents	3/205 (1.5) 2 with acute ALF; 1 with acute-on-chronic ALF	NR	NR	NR	Outpatient visits: Diagnosis made in outpatient setting: 32/205 (15.6) Diagnosis made in inpatient setting: 173/205 (84.4)
Samala 2021 [44]	IN All cases presenting with clinical symptoms and laboratory evidence of hepatitis (elevated liver biochemistries with or without liver biopsy) and positive for HAV IgM antibody (anti- HAV) to IN University Health and Eskenazi Health with HAV from October 2015 to April 2019 (N = 264)	Overall	Ascites: 37/264 (14.0) Ascites in those with underlying cirrhosis: 8/37 (21.6) Ascites in those with underlying CLD without cirrhosis:, 12/37 (32.4) Acute kidney injury: 27/264 (10.0) Acute-on-chronic liver failure in those with underlying cirrhosis: 6/20 (30.0) CLIF-C ACLF grade 1, 1/6 (17.0) CLIF-C ACLF grade 2, 0/6 (0) CLIP-C ACLF grade 3: 5/6 (83.0) Hepatic encephalopathy: 18/264 (7.0) HE in those with underlying cirrhosis: 7/18 (38.9) HE in those with underlying CLD without cirrhosis: 6/18 (33.3) ALF/injury: 9/264 (4.0) 8/9 (88.9) presented with ALF on admission 5/9 (55.5) had underlying CLD	Total deaths: 5/264 (2.0) Deaths related to liver disease: 5/5 (100)	NR	NR	NR	NR
Viray 2019^c^ [45]	HI (Oahu, Big Island, Kauai, Maui) and out of state Outbreak-associated HepA cases reported to Hawaii Department of Health in June to August 2016 (N = 292)	Overall	NR	2/292 (1.0)	74/292 (25.3)	Median (range) d: 3 (1–129)	NR	NR
Wilson 2019 [46]	Kanawha County, WV Outbreak-associated HepA cases reported in WV from January 1 to 28 August 2018 (N = 664)	Overall	NR	1/664 (0.1)	380/664 (57.2)	NR	NR	NR

Abbreviations: ACLF, acute on chronic liver failure; ALF, acute liver failure; ALT, alanine transaminase; AR, Arkansas; AST, aspartate aminotransferase; AZ, Arizona; Bup, buprenorphine; CA, California; CDC, Centers for Disease Control and Prevention; CLD; chronic liver disease; CLIF-C, Chronic Liver Failure Consortium; CO, Colorado; DE, Delaware; ED, emergency department; EMR, electronic medical records; FDA, Food and Drug Administration; FL, Florida; GA, Georgia; GI, gastrointestinal; HAV, hepatitis A virus; HCRU, health care resource utilization; HE, hepatic encephalopathy; HepA, hepatitis A; HI, Hawaii; ICU, intensive care unit; IgM, immunoglobin M; IL, Illinois; IN, Indiana; IV, intravenous; KY, Kentucky; LA, Louisiana; LMPHW, Louisville Metro Department of Public Health and Wellness; MA, Massachusetts; MD, Maryland; MDSS, Michigan Disease Surveillance System, MI, Michigan; MI, myocardial infarction; MN, Minnesota; MS, Mississippi; MSM, men who have sex with men; N, overall population size; n, sample population size; NAC, *N*-acetylcysteine; NAFLD, nonalcoholic fatty liver disease; NC, North Carolina; ND, North Dakota; NIS, National Inpatient Sample; NNDSS, National Notifiable Diseases Surveillance System; NR, not reported; NV, Nevada; NX, naloxone; NY, New York; OH, Ohio; PA, Pennsylvania; SD, standard deviation; SE, standard error; TN, Tennessee; UT, Utah; VA, Virginia; VT, Vermont; WA, Washington; WI, Wisconsin; WV, West Virginia.

^a^Unless otherwise stated.

^b^Unfavorable outcome, defined as occurrence of one or more of the following during hospitalization: death, intensive care admission, acute or chronic liver failure, renal failure, respiratory failure, or shock.

^c^Outbreak(s) occurred solely via food-borne transmission.

^d^National surveillance acute hepatitis A case definition in 2018: acute illness with discrete onset of symptoms consistent with acute viral hepatitis, jaundice, or elevated ALT or aspartate aminotransferase, and IgM antibody to hepatitis A virus (anti-HAV) positive.

^e^Compared with recent incarceration.

^f^HepA outbreak-associated patients who died and whose deaths were determined to be associated with HepA by the respective state health departments.

^g^HepA outbreak-associated patients who had not died.

^h^Of 20 patients who died of HepA-associated causes, 19 (95.0%) had underlying factors (eg, cirrhosis, diabetes, or cardiomyopathy) that may have contributed to increased risk of severe outcomes. Two patients (10.0%) had relapsing HAV infection (defined as recurrent disease within 6 months of last recovery).

^i^One patient with underlying alcoholic cirrhosis–developed GI bleeding and hepatorenal syndrome; 1 had underlying NAFLD; both were considered for transplant. The first patient died and the second patient improved with supportive care.

Of the 33 studies, 30 reported on populations affected by outbreaks, including people: who use drugs or with substance use disorder (SUD) (n = 26), experiencing homelessness (n = 19), currently or recently incarcerated (n = 5), with history of hepatitis B or C infection (n = 4), and MSM (n = 4). Other reported populations affected by HepA outbreaks are listed in Supplementary Table 4.

#### Morbidity

Morbidity-related health outcomes associated with the HepA outbreaks were reported in 13 of 33 studies (Table 1). The most frequently reported morbidities included acute liver failure (ALF)/injury (1.0%–80.4%; reported across n = 6 studies) [33, 36, 39, 40, 43, 44], liver transplantation (0.2%–0.6%; n = 5) [14, 16, 32, 33, 43], hepatic encephalopathy (0.0%–7.0%; n = 4) [18, 40, 41, 44], and sepsis/septic shock (7.9%–43.0%; n = 4) [18, 36, 39, 40], with variability in results observed across study populations. When stratified by survival status, morbidity rates were substantially higher among deceased cohorts than among surviving cohorts, accounting for the wide ranges reported [33, 40].

Other less-commonly reported HepA-related morbidities included acute kidney injury [40, 44], ascites [40, 44], acute-on-chronic liver failure [43, 44], fulminant hepatitis [32, 41], malnutrition [40], cholestatic hepatitis [34], clinically relapsing hepatitis [34], decompensated cirrhosis [41], renal failure, and respiratory failure [35].

#### Mortality

Of 26 out of 33 studies reporting on HepA outbreak-related case fatality, rates ranged from 0%–10.8%, with variability across study populations (Table 1) [16, 26]. According to 4 CDC viral hepatitis surveillance reports, the HepA mortality rate was 0.01 deaths per 100 000 persons in 2016, which increased to 0.02 in 2017, 0.05 in 2018, and 0.04 in 2019, and was higher in men and in people aged ≥55 years [19–22].

Three studies identified factors associated with HepA mortality, including: older age [40, 42]; being White or Asian/Pacific Islander [33, 40]; having certain comorbidities [33, 42]; experiencing homelessness [42]; longer hospital length of stay [33, 40]; hospitalization and intensive care unit (ICU) admission [33]; and higher hospitalization costs [40].

#### Health Care Resource Utilization

Across the SLR, 30 of 33 studies reported HCRU associated with HepA, including any hospitalization (n = 29), ICU admission (n = 7), 30-day readmission rates (n = 2), outpatient visits (n = 1), and medication/treatment utilization (n = 1). Full HCRU results are listed in Table 1. Of studies reporting any hospitalization in the overall population due to outbreaks that did not solely occur via food-borne transmission (n = 17), the majority (n = 14) reported hospitalization rates greater than 50%, ranging from 41.6% of HepA cases nationally reported to the CDC [22], to 84.8% in a cross-sectional study of HepA cases in Michigan [32]. One study found that the large majority of HepA diagnoses were made in the inpatient (84.4%) versus outpatient (15.6%) setting [43]. Reichenbach et al (2021) suggested that this was likely due to educational campaigns by the city's health department about the ongoing outbreak, which may have encouraged patients with symptoms to seek out evaluation, while diagnoses in the outpatient setting were likely often among asymptomatic patients [43].

Length of stay, reported in 10 studies, ranged in the overall population from a mean of 2.5 days among hospitalized patients (predominantly people with SUD) in Charleston, West Virginia [18] to 6 days in hospitalized patients (predominantly people with SUD and/or experiencing homelessness) in San Diego, California [38]. ICU admission rates ranged from 6.3% in West Virginia in 2016–2019 [32], to 26.5% in Southeast Michigan from 2016–2017 [16]. According to a 2016–2019 case-control study in Kentucky, Michigan, and West Virginia, 83.5% of fatal HepA cases were admitted to the ICU, while only 11.1% of HepA survivors were admitted [33]. Two studies identified 30-day readmission rates of 4.2% and 23.4% among HepA patients presenting to health systems in Southeast Michigan and San Diego, California, respectively [16, 39]. No studies were identified that reported on outpatient visit and emergency department visit rates and treatment utilization in the overall population; however, 1 San Diego, California study on treatment utilization in a high-risk (ALF) population found significantly higher rates of treatment utilization in ALF versus non-ALF groups [36].

### Economic Burden

In total, 10 studies reported on the economic burden of HepA outbreaks, spanning direct medical costs associated with HCRU and public health intervention costs (Table 2). These studies focused on local or state outbreak data (n = 6) and national data (n = 4), and 7 reported on populations affected by the outbreaks, including people who use drugs/people with SUD (n = 5), people experiencing homelessness (n = 3), food service workers (n = 2), and MSM (n = 1). One study reported person-to-person transmission, while among the remainder, mode of transmission was not reported or not applicable to the study design.

**Table 2.** Costs Associated With the US HepA Outbreaks

Reference	Location Study Population (N Cases)	Study Period	Currency Year	Cost Definition	Cost ($)
Direct medical costs					
Batdorf 2021 [47]	WV WV Medicaid beneficiaries with a primary or secondary diagnosis of HepA on a medical claim during 1 January 2018–31 July 2019 (N = 1989)	1 January 2018–31 July 2019	NR	Overall HepA-related Medicaid direct clinical costs	$5 668 729
				SUD HepA-related Medicaid direct clinical costs	$4 390 027
				Overall inpatient hospital claims with diagnosis of HepA and disorder of liver	$1 440 907
				SUD inpatient hospital claims with diagnosis of HepA and disorder of liver	$1 025 389
Dankwa 2021 [26]	Louisville, KY HepA cases captured by surveillance conducted by the LMPHW (N = 501)	September 2017–June 2019	NR	Hospitalization costs averted by vaccination program	$490 000 (95% CI, $310 000–700 000)
Hofmeister 2020 [14]	National All HepA-related hospitalizations captured in the 2017 Healthcare Cost and Utilization Project NIS (Sample size NR)	2017	2017	Nationwide average cost per HepA-related hospitalization	$16 232 (SD, $602; 95% CI, $15 052–$17 411)
				West North Central Census Division (lowest average cost)	$12 921 (SD, $1443; 95% CI, $10 091–$15 750)
				Pacific Census Division (highest average cost)	$19 680 (SD, $1932; 95% CI, $15 891–$23 467)
		July 2016-February 2020	NR	Total HepA-related hospitalization cost nationally	$306.8 million (SD, $11.4 million)
Lee 2020 [40]	National Hospitalized patients identified from the 2011 to 2017 NIS with acute HepA infection stratified by survival status (N = 11 740)	2011–2017	NR	Inpatient costs of deceased	$155 523
				Inpatient costs of survivors	$48 611
Wilson 2020 [48]	Local health department area (NR) NA (model study); data from local health department (program costs), Ozawa 2016 [49] (outpatient, inpatient, self-care costs), Luyten 2009 (public health/prevention costs in case of outbreak)	1 October 2015–30 September 2016	2016	Inpatient costs [49]	$15 562
				Outpatient costs [49]	$474 (adjusted)
				Costs with no care [49]	$0
Public health intervention costs					
Baum 2020 [50]	New York City, NY NA; cost of collaboration of DOHMH with private clinic network in 2017 compared to cost of emergency activation in 2015 for HepA public health response for restaurant employees (2015, N = 276; 2017, N = 201)	2015	2017	Total cost of 2015 DOHMH emergency activation	$65 831
				DOHMH personnel services total costs	$55 854 (84.8% of total cost)
				Nonovertime costs	$30 569 (46.4%)
				Cash overtime costs	$23 025 (35.0%)
				Compensatory time	$2260 (3.4%)
				HepA vaccine	$6778 (10.3%)
				Emergency activation miscellaneous	$140 (0.2%)
				Cost per restaurant employee evaluated	$238
		2017	2017	Total cost of 2017 collaboration with private clinic network	$50 914
				DOHMH personnel services total costs	$3146 (6.2% of total cost)
				Nonovertime costs	$2878 (5.7%)
				Cash overtime costs	$118 (0.2%)
				Compensatory time	$15 (0.3%)
				HepA vaccine	$5481 (10.8%)
				Transportation of vaccine	$50 (0.1%)
				Private clinic network total costs	$42 238 (83%)
				Personnel services	$38 080 (74.8%)
				HAV IgM antibody tests	$3366 (6.6%)
				Medical supplies	$792 (1.6%)
				Cost per restaurant employee evaluated	$253
Duncan 2018 [51]	San Diego County, CA Adults receiving HepA vaccines at FHCSD clinics between the week ending 14 August 2017 and the week ending 5 March 2017 (N = 7521)	2017	NR	Cost of vaccine intervention using CDC's cost list at $25.73 per dose	$193 515
Ghildayal 2019 [52]	National NA; dynamic transmission model with 2 scenarios: 1 with a universal vaccination policy, and 1 without a universal vaccination policy	2009–2028	NR	Accrued cost (total; no vaccination policy)	$2 678 008 491
				Accrued hospital cost (total; no vaccination policy)	$1 919 964 995
				Accrued vaccine cost (total; no vaccination policy)	$758 043 495
				Accrued cost (total, universal vaccination policy)	$10 552 726 375
				Accrued hospital cost (universal vaccination policy)	$181 442 563
				Accrued vaccine cost (universal vaccination policy)	$10 371 283 812
				Vaccination cost for 2 doses (universal vaccination policy)	$60
Kaigh 2020 [37]	Philadelphia, PA All ED visits that were evaluated for HepA before and after a vaccination program from 16 July 2019 to 8 October 2019 (prevaccination program: 67 admitted, 73 diagnosed with HepA; postvaccination program: 31 admitted, 38 diagnosed with HepA)	July 16–8 October 2019	NR	HepA vaccine dose	$58.40 (free for intervention)
Schwartz 2022 [53]	NA/National Facebook advertisement campaign for target audiences (MSM and FSW) from 30 October 2019 to 6 November 2019	30 October 2019–6 November 2019	NR (likely 2019)	Overall audience ad	$128.21 ($0.97 per result)
				MSM ad 1	$13.75 ($0.36 per result)
				MSM ad 2	$108.04 ($0.93 per result)
				FSW ad 1	$144.47 ($0.99 per result)
				FSW ad 2	$51.21 ($0.90 per result)
Wilson 2020 [48]	Local health department area (NR) NA (model study); data from local health department (program costs), Ozawa 2016 (outpatient, inpatient, self-care costs), Luyten 2009 (public health/prevention costs in case of outbreak)	1 October 2015–30 September 2016	2016	HepA vaccine administration program costs	$10 188 000
				HepA adult immunization program costs	$103 004
				HepA vaccine dose unit cost	$27.82
				Nonvaccine program cost per dose	$40.10
				Nonvaccine program costs total (for local health department and community partners providing range of vaccines including HepA)	$377 625
				Staffing, local health department	$335 437
				Staffing, community partners	$10 400
				Travel	$3780
				Equipment	$4007
				Supplies, medical	$15 000
				Supplies, office	$3000
				Technology	$3000
				Printed materials	$3000
				Public health/prevention costs in case of an outbreak (per case)	$592 (Luyten 2009)

Abbreviations: CA, California; CDC, Centers for Disease Control and Prevention; CI, confidence interval; DOHMH, Department of Health and Mental Hygiene; ED, emergency department; FHCSD, Family Health Centers of San Diego; FSW; food service workers; HAV, hepatitis A virus; HepA, hepatitis A; IgM, immunoglobin M; KY, Kentucky; LMPHW, Louisville Metro Public Health and Wellness; MSM, men who have sex with men; NA, not applicable; NIS, National Inpatient Sample; NR, not reported; NY, New York; PA, Pennsylvania; SD, standard deviation; SUD, substance use disorder; WV, West Virginia.

#### Direct Medical Costs Associated With Health Care Resource Utilization

Five studies reported on direct medical costs associated with HCRU, such as hospitalization, outpatient, vaccination, and Medicaid-specific direct clinical costs. In a 2017 national cohort study, the estimated nationwide average cost per HepA-related hospitalization was $16 232 (ranging from $12 921 to $19 680 by region; 2017 USD) [14]. Another study in a local health department area reported that estimated inpatient and outpatient costs per HepA case in 2016 were $15 562 and $474, respectively (2016 USD) [48]. A national database review reporting on acute HepA cases from 2011–2017 reported estimated inpatient costs stratified by survival status, which ranged from $48 611 per surviving patient to $155 523 per deceased patient (USD, currency year not reported [NR]) [40]. One study in West Virginia identified the economic burden related to direct clinical and inpatient costs of the HepA outbreaks among people with SUD, reporting that people with SUD incurred higher costs than people without SUD (ie, 77% of Medicaid direct clinical costs; 71% of total inpatient costs) from 2018–2019 [47]. Total HepA-related hospitalization costs from 2016–2020 were estimated to be approximately $306.8 million (USD, currency year NR) [14].

#### Public Health Intervention Costs

Six studies reported public health intervention costs, centered around prevention (vaccination costs, as well as advertising, administrative, staffing, and equipment costs). In 2017, the cost of the New York City Department of Health and Mental Hygiene's emergency activation collaboration, offering HepA testing and vaccines to food service workers, was $50 914 ($253 per restaurant employee evaluated; 2017 USD) [51]. One national study also evaluated public health intervention costs in a high-risk population (MSM), in addition to food service workers, reporting the cost of a 2019 8-day national Facebook advertising campaign that reached 53 422 users, with an average cost-per-link-click of $0.92 and total amount spent of $445.68 (USD, currency year NR) [54].

Two cost-effectiveness analyses reporting HepA outbreak-related public health intervention costs were also identified, 1 of which modeled total costs of a 2015–2016 HepA vaccination program at $10 188 000 per 100 000 clients (2016 USD, currency year NR) [48]. The other national model found that a universal HepA vaccination policy in the United States was cost-effective at $55 778 per quality-adjusted life year gained (USD, currency year NR), compared to a willingness-to-pay threshold of $100 000/quality-adjusted life year gained, and resulted in lower HepA incidence [53].

### Outbreak Management

**Figure 2.**
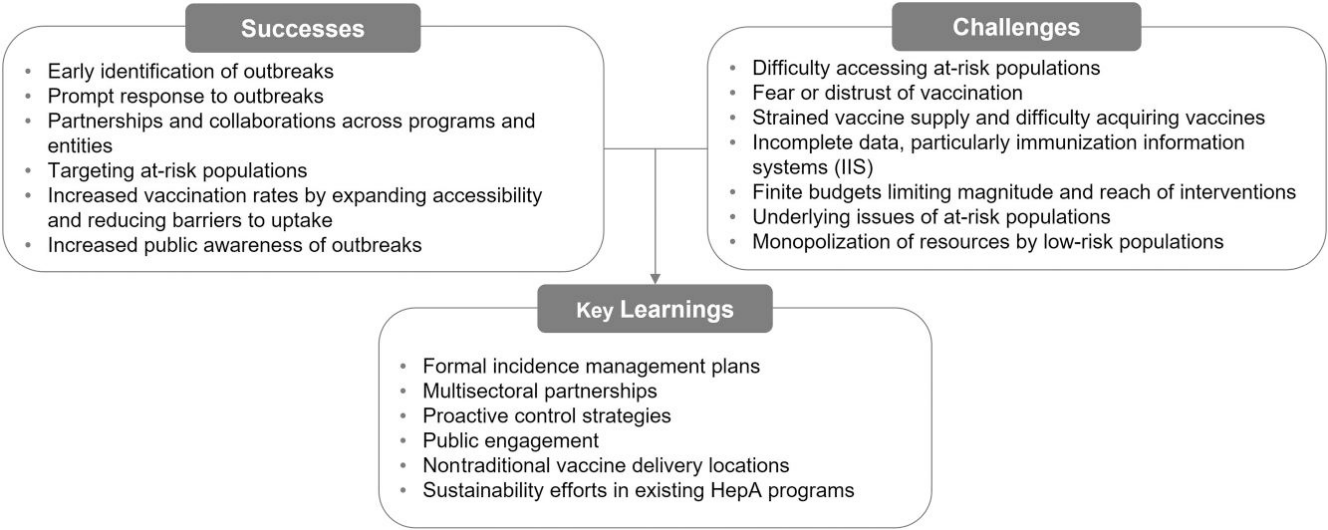
Successes, challenges, and key learnings associated with US hepatitis A (HepA) outbreak management.

The TLR included 34 studies reporting on the successes (n = 25), challenges (n = 28), and key learnings (n = 24) of outbreak management efforts related to ongoing HepA outbreaks (Supplementary Table 5 and Figure 2). No studies reporting cost drivers of outbreak management were identified.

Most of these studies reported on HepA cases at the local or state level (n = 21), rather than multistate (n = 5) or national or global level (n = 8), and many reported on some form of vaccination-related intervention, followed by education and hygiene interventions. Overall, 27 of 34 studies reported on populations affected by the HepA outbreak, including people experiencing homelessness (n = 20), people who use drugs or with SUD (n = 15), people currently or recently incarcerated (n = 6), MSM (n = 5), and people who ate contaminated food (n = 3). Study design and patient populations included in the TLR are summarized in Supplementary Table 6.

#### Successes

Early detection, early preparation, and prompt response were all found to have a considerable positive impact on HepA outbreak management, as evidenced in California's early outbreak detection efforts, which led to increased vaccination in vulnerable populations [55], Tennessee's allocation of vaccination funds during early outbreak stages [56], and Massachusetts’ integration of surveillance data to expedite outbreak response [57].

Partnerships between organizations were also a key success. Coordination between public health agencies, hospitals, and pharmacies aided in containing a San Diego, California outbreak by increasing vaccination [38, 52]. In Michigan, partnerships expanded the reach of vaccine and education materials to at-risk populations in locations such as bars, prisons, and shelters [58].

Outbreak management tools that used an individualized approach to target at-risk populations were also successful, especially in reducing barriers to vaccination uptake. These tools included targeted computerized alerts [59]; efforts to engage medically underserved populations, implementing innovative vaccine delivery strategies, vaccine tracking, repeat community vaccination events, and vaccination safety informational materials [60]; and offering vaccines in nontraditional settings such as jails, SUD treatment programs, and homeless services [61, 62]. For example, one 2017 San Diego, California outbreak response reported success in adopting a highly individualized approach using vaccination events, educational outreach, and sanitation campaigns (eg, handwashing stations, increased access to public restrooms/hygiene kits) targeted towards people experiencing homelessness [63].

Finally, increasing overall public awareness (eg, through social media) was found to be an effective measure mitigating further HepA outbreak spread (Supplementary Table 5 and Figure 2) [54, 55, 64].

#### Challenges

Vaccine hesitancy due to fear and distrust, and difficulty accessing at-risk populations, were both challenges of implementing outbreak management tools. In multiple studies, individuals who hesitated or refused HepA vaccination expressed beliefs including danger or uselessness of the vaccine and mistrust of vaccinators or manufacturers [52, 65], as well as of public officials and health care providers (HCPs) [66]. Limited access to medical care or vaccination sites [38, 62], geographic constraints [61], limited resources within correctional facilities [31], and gender discrepancies in social media usage also contributed to difficulty reaching at-risk groups [54].

Other challenges included strains in vaccination supply and resource requirements during periods of high demand and monopolization, and/or scarcity of available resources [52, 67–71]. One San Diego, California study found that because media coverage tends to better reach populations who are not at high risk for HepA, those populations might seek resources that should be preserved for at-risk individuals [52].

Incomplete data on HepA vaccination rates was described as a challenge, as reporting HepA vaccination in immunization information systems is not required in some states [72]. Limited funding was also commonly reported, with supplies, vaccines, and storage equipment further constrained during the coronavirus disease 2019 (COVID-19) pandemic [26, 56, 57, 66, 73, 74]. Additional challenges in outbreak management among at-risk populations were observed at a more systemic level. For example, poor sanitation and/or homelessness have been identified as root causes of outbreaks that need to be addressed (Supplementary Table 5 and Figure 2) [37, 55, 63, 75].

#### Key Learnings

Formal incidence management plans were found to improve coordination and communication across affected agencies, especially when collaborations were established in advance of outbreaks [55, 60, 61, 66, 68]. Proactive control strategies, particularly vaccination among high-risk groups before or in early stages of outbreaks, were also effective [17, 75].

Key learnings also included the importance of understanding the root causes of disparities in HepA vaccination among different groups. A retrospective cohort study in Illinois and Wisconsin found that among HepA vaccine recipients, people who were insured with Medicare, Hispanic, non-Asian, or who had a history of incarceration were significantly less likely to be vaccinated [76]. Furthermore, directly addressing root causes [75], such as barriers to sanitation and vaccination among people experiencing homelessness [65, 77], is necessary. Alternative routes, such as needle exchange programs [17], and improving hygiene facility and supply accessibility [52], could also be utilized to decrease HepA spread.

Due to distrust of vaccination and government health policies among high-risk groups, developing strategic, nontraditional ways of reaching these groups and communicating HepA vaccination safety and importance were necessary. This included targeted, accessible outreach on social media platforms and word-of-mouth via community leaders [68]. Collaborations, especially those strengthening partnerships between organizations with existing trusted relationships with vulnerable populations, could also improve vaccination uptake [60]. Implementing nontraditional vaccine delivery locations that might be more convenient for higher-risk groups, such as jails and SUD facilities, could also increase vaccination access for these groups [31, 73].

Other key learnings included the importance of building upon preexisting materials or data [26, 78], as well as using informatics tools in increasing vaccination efforts [59], positive public perception of public health efforts [67], formal record keeping [45, 72], and improved social media messaging [54] (Supplementary Table 5 and Figure 2).

## DISCUSSION

This review demonstrates the considerable burden of HepA on health and economic outcomes, particularly among at-risk populations. By implementing the successes and addressing the challenges of past HepA outbreak responses, the morbidity, mortality, associated HCRU, and economic burden of these outbreaks can be reduced. Increased vaccine awareness, education, and uptake through targeted efforts of HCPs, public health department leaders, and community organizations are key in preventing future HepA outbreaks.

National and international initiatives have been enacted to prevent and eliminate HepA, including the Viral Hepatitis National Strategic Plan (2021–2025) in the United States and the World Health Organization's Global Health Sector Strategy on Viral Hepatitis [79, 80]. However, vaccination coverage in the United States remains low, especially among populations at highest risk of HepA infection, such as injection drug users and MSM [81]. Therefore, it is critical to address key challenges that remain around vaccination distrust, knowledge gaps, and disease misinformation, via education strategies targeting high-risk groups.

In addition, although HepA and HepA & HepB combination vaccines are available and recommended for adults by the ACIP, adherence to and knowledge of ACIP guidelines vary, and some HCPs still report not recommending vaccination against HepA for reasons including low perceived risk of certain patient populations and uncertainty of guidelines [82, 83]. Focusing on prevention, including targeted efforts in HCP education, could be beneficial in increasing vaccine uptake and reducing HepA disease burden. For example, educating HCPs on risk factors, and making HCPs aware that the ACIP recommends that any person who was not vaccinated previously may be vaccinated, may help to prevent infection among at-risk populations who experience stigma (eg, persons experiencing homelessness, drug users, MSM) [81]. Given the risk of hospitalization and associated direct medical and indirect costs of HepA infection, vaccinating patients without confirmed risk factors may help to further decrease the HCRU and economic burden of HepA. Ultimately, the key outbreak management considerations identified in this study can be used to inform future outbreaks across diverse disease areas outside of HepA, especially other vaccine-preventable diseases.

Among this study's limitations, small sample sizes and variations in study design, populations, and outcomes of included studies, as well as the review's scope (ie, limited to US outbreaks), could impact the generalizability of SLR findings. Gaps in available literature, such as the lack of studies reporting direct and indirect medical costs associated with HepA health outcomes, were also a limitation. The scope also did not include an evaluation of the COVID-19 pandemic's impact on the health outcomes and economic burden of the HepA outbreaks, such as altered health-seeking behaviors and diverted public health resources. There is also a systemic underreporting of HepA cases, especially asymptomatic or nonhospitalized cases, which likely underestimates the true burden of HepA disease and consequently overestimates per-person costs of infection. Differences in ICD coding to define HepA-related outcomes may have also led to variability in reported direct medical costs associated with HCRU. Limitations inherent to conducting TLRs included the possibility of noncomprehensive study identification and reviewer bias. As evidence synthesis focused only on studies considered most relevant, not all evidence was covered in detail in this review; however, key evidence addressing the objectives were identified and included where available. Future research should explore the direct and indirect costs of HepA health outcomes, as well as the effect of the COVID-19 pandemic on HepA incidence and related care.

## CONCLUSIONS

This review highlighted the substantial clinical and economic burden of recent HepA outbreaks among US adults, as well as the successes, challenges, and key learnings of outbreak management. By implementing successful practices more broadly and addressing the challenges of past HepA outbreak responses, the considerable morbidity, mortality, associated HCRU and economic burden of these outbreaks can be reduced. Increasing adult vaccination rates via targeted efforts of HCPs and community organizations is essential for preventing future outbreaks. The key learnings identified in this review may help inform local and national decision makers on how to best facilitate and support approaches to reducing HepA burden, as well as the management of outbreaks in other disease areas. Ultimately, these results support the ongoing need for prevention measures such as adult vaccination, especially among high-risk populations, to lower the burden of HepA.

## Supplementary Data

Supplementary materials are available at *The Journal of Infectious Diseases* online (http://jid.oxfordjournals.org/). Supplementary materials consist of data provided by the author that are published to benefit the reader. The posted materials are not copyedited. The contents of all supplementary data are the sole responsibility of the authors. Questions or messages regarding errors should be addressed to the author.

## Supplementary Material

jiae087_Supplementary_Data

## Notes

***Acknowledgments.*** The authors acknowledge Sneha Kelkar, OPEN Health Group, Bethesda, USA for contributions to study conceptualization, and Seongbin Shin, GSK, Philadelphia, USA for publication management. The authors also thank Costello Medical for editorial assistance and publication coordination, on behalf of GSK, and acknowledge Océane Parker, Costello Medical, Boston, USA for medical writing and editorial assistance based on authors’ input and direction.

***Authors contributions.*** All authors made substantial contributions to study conception and design, and analysis and interpretation of the data; drafted the article or revising it critically for important intellectual content; and gave final approval of the version of the article to be published.

***Data sharing.*** The datasets generated during and/or analyzed during the current study are available from the corresponding author on reasonable request.

***Financial support*.** This work was supported by GSK (Study identifier eTrack HE_VEO-000402). Support for third-party writing assistance for this article, provided by Océane Parker, Costello Medical, Boston, USA was funded by GSK in accordance with Good Publication Practice guidelines (https://www.ismpp.org/gpp-2022). Funding to pay the Open Access publication charges for this article was provided by GSK.
