# Supplementary material for: The Burden of Hepatitis A Outbreaks in the United States: Health Outcomes, Economic Costs, and Management Strategies
Source: J Infect Dis. 2024 Feb 28;230(1):e199–218. doi: 10.1093/infdis/jiae087 (PMC11272058; doi:10.1093/infdis/jiae087)
Supplement: jiae087_Supplementary_Data [file jiae087_supplementary_data.docx]

**SUPPLEMENTARY MATERIALS**

**Table of Contents**

**Supplementary Table 1.** SLR MEDLINE/Embase (via ProQuest) search strategy

**Supplementary Table 2.** TLR MEDLINE/Embase (via ProQuest) search strategy

**Supplementary Table 3.** PICOS study eligibility criteria for inclusion in the SLR

**Supplementary Table 4.** Study design and patient population of studies included in the SLR

**Supplementary Table 5.** Outbreak management considerations of studies included in the TLR

**Supplementary Table 6.** Study design and patient population of studies included in the TLR

**Supplementary Table 1.** SLR MEDLINE/Embase (via ProQuest) search strategy

| **Criteria** | **#** | **Search Terms** | **Hits** |
| --- | --- | --- | --- |
| Disease | S1 | ("Hepatitis A" OR "Hepatitis-A" OR "HEP-A" OR "HEP A" OR "Hep A" OR "Hep-A" OR "Hepatitis A virus" OR "HAV outbreak" OR "HAV") OR EMB.EXACT("Hepatitis A") OR EMB.EXACT.EXPLODE("Hepatitis A") OR MESH.EXACT("Hepatitis A") OR MESH.EXACT.EXPLODE("Hepatitis A") | 70,023 |
| Key terms of interest | S2 | TI,AB("illicit drug use" OR "illicit drug-use" OR "drug use" OR "drug-use" OR "food-borne" OR "foodborne" OR "person-to-person" OR "incarceration" OR "incarcerated" OR "Men Who Have Sex with Men" OR "MSM" OR "homeless" OR "homelessness" OR "unstable housing" OR “housing insecurity” OR “housing instability” OR “unhoused” OR "outbreak" OR "outbreaks" OR "United States" OR "U.S." OR "US") | 2,465,929 |
| Direct Cost | S3 | MESH.EXACT("Economics" OR "Value of Life" OR "Economics, Nursing" OR "Economics, Pharmaceutical") OR MESH.EXACT.EXPLODE("Costs and Cost Analysis" OR "Economics, Hospital" OR "Economics, Medical" OR "Fees and Charges" OR "Budgets" OR "Models, Economic" OR "Decision Theory") OR EMB.EXACT("socioeconomics" OR "economic aspect" OR "financial management" OR "economics" OR "economic model" OR "cost" OR "budget" OR "Monte Carlo method" OR "Markov chain" OR "decision theory" OR "decision tree") OR EMB.EXACT.EXPLODE("health economics") OR TI,AB(fiscal OR funding OR economic* OR cost* OR price OR prices OR pricing OR pharmacoeconomic OR pharmaco-economic OR expenditure OR expenditures OR expense OR expenses OR financ* OR (value NEAR/2 (money OR monetary)) OR markov OR "monte carlo") OR TI,AB(decision NEAR/2 (tree OR analy* OR model OR models)) | 4,199,267 |
| Indirect cost | S4 | MESH.EXACT("Employer Health Costs" OR "Efficiency" OR "Presenteeism" OR "Absenteeism") OR MESH.EXACT.EXPLODE("Salaries and Fringe Benefits") OR EMB.EXACT("productivity" OR "medical leave" OR "presenteeism" OR "absenteeism") OR EMB.EXACT.EXPLODE("salary and fringe benefit") OR TI,AB(absenteeism OR presenteeism OR ((productivity OR productive OR sick OR work OR sickness OR disability OR employee OR employer) NEAR/3 (limitations OR impact OR day OR days OR leave OR absence OR absences OR incapacity OR loss OR lost OR losing)) OR "loss-of-work") OR TI,AB((caregiver OR carer OR family OR familial) near/3 burden) | 290,198 |
| Health outcomes | S5 | (EMB.EXACT.EXPLODE("health care utilization" OR "hospital care" OR "long term care") OR EMB.EXACT("hospital admission" OR "medical specialist" OR "hospital patient" OR "outpatient")) OR (TI,AB((resource OR healthcare OR "health care") NEAR/5 (use OR utilization OR utilisation)) OR TI,AB((visit OR visits OR admission OR admissions OR readmission OR readmissions OR re-admission OR re-admissions OR stay OR stays OR stayed OR day OR days) NEAR/3 (physician OR emergency OR specialist OR outpatient OR inpatient OR "primary care" OR "length of stay" OR practitioner OR hospital OR clinic OR "long-term care" or "long term care" OR “urgent care” OR “intensive care unit” OR “intensive treatment unit” OR “intensive therapy unit” OR “critical care unit” OR “ICU” OR “CCU”)) OR TI,AB(hospitalization OR hospitalizations OR "length of stay" OR "in home care" OR "in-home care" OR "mortality" OR sequela* OR complication)) OR EMB.EXACT.EXPLODE("vaccination reaction") OR EMB.EXACT("adverse event") OR MESH.EXACT("Drug-Related Side Effects and Adverse Reactions") | 8,369,695 |
| Location – United States | S6 | EMB.EXACT.EXPLODE("United States") OR MESH.EXACT.EXPLODE("United States") OR TI,AB(“United States” OR “US” OR America*) OR AF(“United States” OR “USA” OR “US” OR "AL" OR "AK" OR "AZ" OR "AR" OR "CA" OR "CO" OR "CT" OR "DE" OR "DC" OR "FL" OR "GA" OR "HI" OR "ID" OR "IL" OR "IN" OR "IA" OR "KS" OR "KY" OR "LA" OR "ME" OR "MD" OR "MA" OR "MI" OR "MN" OR "MS" OR "MO" OR "MT" OR "NE" OR "NV" OR "NH" OR "NJ" OR "NM" OR "NY" OR "NC" OR "ND" OR "OH" OR "OK" OR "OR" OR "PA" OR "RI" OR "SC" OR "SD" OR "TN" OR "TX" OR "UT" OR "VT" OR "VA" OR "WA" OR "WV" OR "WI" OR "WY" OR "Alabama" OR "Alaska" Or "Arizona" Or "Arkansas" Or "California" Or "Colorado" Or "Connecticut" Or "Delaware" Or "District Of Columbia" Or "Florida" Or "Georgia" Or "Hawaii" Or "Idaho" Or "Illinois" Or "Indiana" Or "Iowa" Or "Kansas" Or "Kentucky" Or "Louisiana" Or "Maine" Or "Maryland" Or "Massachusetts" Or "Michigan" Or "Minnesota" Or "Mississippi" Or "Missouri" Or "Montana" Or "Nebraska" Or "Nevada" Or "New Hampshire" Or "New Jersey" Or "New Mexico" Or "New York" Or "North Carolina" Or "North Dakota" Or "Ohio" Or "Oklahoma" Or "Oregon" Or "Pennsylvania" Or "Rhode Island" Or "South Carolina" Or "South Dakota" Or "Tennessee" Or "Texas" Or "Utah" Or "Vermont" Or "Virginia" Or "Washington" Or "West Virginia" Or "Wisconsin" Or "Wyoming") | 29,726,965 |
| Combined | S7 | (S1 AND S2) OR (S1 AND (S3 OR S4 OR S5)) | 16,791 |
| Filter–Human Studies only | S8 | EMB.EXACT.EXPLODE(animal) OR MESH.EXACT.EXPLODE(Animals) | 56,786,725 |
|  | S9 | EMB.EXACT.EXPLODE(human) OR MESH.EXACT.EXPLODE(Humans) | 46,060,041 |
|  | S10 | S8 NOT (S8 AND S9) | 10,726,788 |
|  | S11 | S7 NOT S10 | 16,275 |
| **Conference abstracts of interest** | **S12** | **S6 AND S11 AND PD(>2015) AND RTYPE(“conference abstract”) AND CF(“american college of gastroenterology” OR “AMCP” OR “academy of managed care pharmacy” OR “ISPOR” OR “infectious diseases week” OR AASLD) AND LA(English)** | 248^a^ |
| **Full-texts of interest** | **S13** | **S6 AND S11 AND PD(>2015) AND LA(English) NOT RTYPE(“conference abstract”)** | 1,535^a^ |
| **Combined** | **S14** | **S12 OR S13** | 1,783^a^ |

^a^ Duplicates are removed from the search and from the result count.

Abbreviations: SLR, systematic literature review.

**Supplementary Table 2.** TLR MEDLINE/Embase (via ProQuest) search strategy

| **Criteria** | **#** | **Search Terms** | **Hits** |
| --- | --- | --- | --- |
| HepA | S1 | (“Hepatitis A” OR “Hepatitis-A” OR “HEP-A” OR “HEP A” OR “Hep A” OR “Hep-A” OR “Hepatitis A virus” OR “HAV outbreak” OR “HAV”) OR EMB.EXACT(“Hepatitis A”) OR EMB.EXACT.EXPLODE(“Hepatitis A”) OR MESH.EXACT(“Hepatitis A”) OR MESH.EXACT.EXPLODE(“Hepatitis A”) | 70,023 |
| TLR terms of interest | S2 | EMB.EXACT(“public health campaign”) OR EMB.EXACT(“public service announcement”) OR MESH.EXACT.EXPLODE(“Public Health Administration”) OR mesh(“Disease Outbreaks–prevention & control”) OR TI(guidelines OR guidance OR control OR manage* OR response OR “public health” OR “opportunity cost” OR public near/3 cost OR government OR administration OR department OR vaccination) | 4,245,637 |
| US | S3 | EMB.EXACT.EXPLODE(“United States”) OR MESH.EXACT.EXPLODE(“United States”) OR TI,AB(“United States” OR “US” OR America*) OR AF(“United States” OR “USA” OR “US” OR “AL” OR “AK” OR “AZ” OR “AR” OR “CA” OR “CO” OR “CT” OR “DE” OR “DC” OR “FL” OR “GA” OR “HI” OR “ID” OR “IL” OR “IN” OR “IA” OR “KS” OR “KY” OR “LA” OR “ME” OR “MD” OR “MA” OR “MI” OR “MN” OR “MS” OR “MO” OR “MT” OR “NE” OR “NV” OR “NH” OR “NJ” OR “NM” OR “NY” OR “NC” OR “ND” OR “OH” OR “OK” OR “OR” OR “PA” OR “RI” OR “SC” OR “SD” OR “TN” OR “TX” OR “UT” OR “VT” OR “VA” OR “WA” OR “WV” OR “WI” OR “WY” OR “Alabama” OR “Alaska” Or “Arizona” Or “Arkansas” Or “California” Or “Colorado” Or “Connecticut” Or “Delaware” Or “District Of Columbia” Or “Florida” Or “Georgia” Or “Hawaii” Or “Idaho” Or “Illinois” Or “Indiana” Or “Iowa” Or “Kansas” Or “Kentucky” Or “Louisiana” Or “Maine” Or “Maryland” Or “Massachusetts” Or “Michigan” Or “Minnesota” Or “Mississippi” Or “Missouri” Or “Montana” Or “Nebraska” Or “Nevada” Or “New Hampshire” Or “New Jersey” Or “New Mexico” Or “New York” Or “North Carolina” Or “North Dakota” Or “Ohio” Or “Oklahoma” Or “Oregon” Or “Pennsylvania” Or “Rhode Island” Or “South Carolina” Or “South Dakota” Or “Tennessee” Or “Texas” Or “Utah” Or “Vermont” Or “Virginia” Or “Washington” Or “West Virginia” Or “Wisconsin” Or “Wyoming”) | 29,704,635 |
| Combination | S4 | S1 AND S2 AND S3 AND PD(>2015) NOT RTYPE(“conference abstract”) | Approx. 600 |

Abbreviation: TLR, targeted literature review.

**Supplementary Table 3.** PICOS study eligibility criteria for inclusion in the SLR

|  | **Inclusion Criteria** | **Exclusion Criteria** |
| --- | --- | --- |
| **Population** | - Adults ≥18 years of age in US living in an area with a HepA outbreak | - Children or adolescents <18 years of age - Population not identified or not specific to US |
| **Intervention** | Not applicable | Not applicable |
| **Comparator** | Not applicable | Not applicable |
| **Outcomes** | Health outcomes associated with HepA outbreaks:   - HepA-related complications and sequalae - Morbidity (e.g., complicated HepA, cholestatic hepatitis, ALF/injury, liver transplant) - Mortality - Vaccination-related AEs - Hospitalizations and LOS (including ICU) - Outpatient services (e.g., primary care, urgent care, visit to specialist) - ED visits - Ancillary care - Medication use and other treatment use   Economic burden associated with HepA outbreaks:   - Direct costs (e.g., hospitalization, outpatient, ED, medication and other treatment, non-medical, and other resource utilization) - Indirect costs (e.g., absenteeism, presenteeism, productivity loss [including non-market productivity loss], caregiver burden) - Public health intervention costs (e.g., screening, vaccine acquisition and administration, disease awareness campaigns, public notifications) | - Studies reporting outcomes that are not clearly associated with recent HepA outbreak from 2016–present - Only reporting outcomes not included in the inclusion criteria |
| **Study design** | - Observational studies^a^ (e.g., cohort, case-control, case series) - Systematic reviews^b^ - Modelling studies^b^ | - Editorials, notes, letters - Non-systematic reviews - Interventional trials (randomized or single-arm) - Case reports or case series (<5 sample size) |
| **Other** | - Articles published in 2016 or later - Key conference abstracts indexed in Embase and published in 2016 or later | - Non-English language - Animal studies |

^a^ Includes reports from health agencies and other sources that report outcomes of interest obtained through the targeted literature search.

^b^ For identification of studies via the bibliography.

Abbreviations: AE, adverse event; ALF, acute liver failure; ED, emergency department; HepA, Hepatitis A; ICU, intensive care unit; LOS, length of stay; PICOS, Population, Intervention, Comparison, Outcome, Study design; SLR, systematic literature review; US, United States.

**Supplementary Table 4.** Study design and patient population of studies included in the SLR

| **Reference** | **Study dates** | **Study design** | **Data source** | **Population primarily affected by outbreak** | | | | |
| --- | --- | --- | --- | --- | --- | --- | --- | --- |
|  |  |  |  | **Age (as reported)** | **Males**  **(N [%])** | **Race/ethnicity**  **(N [%])** | **Comorbidities**  **(N [%])** | **Risk factors**  **(N [%])** |
| Altamimi 2018 [16] | August 2016–December 2017 | Retrospective chart review | EMR | Mean: 51 years | NR (54%) | NR | Diabetes, NR (18.6%) | Illicit drug use, NR (23%) Alcohol abuse, NR (22%) Travel outside the state within 2 weeks prior to diagnosis, NR (3%) Food handler, NR (4.2%) |
| Brouwer 2020 [17] | August 2016–December 2018 | Disease transmission model | MDSS | Median: 40 years | 594 (65%) | NR | History of HepB or C, 233 (26%) | Substance use, 427 (47%)  Non-IV only, 172 (19%) IV only, 71 (8%) Both, 159 (17%)  Homeless/transient housing, 114 (13%) Institutional risk, 100 (11%) MSM, 79 (9%) |
| Butt 2020 [18] | March 2017–November 2018 | Retrospective chart review | Patient records at tertiary care center | Mean (SD): 42 (13.3) years | 291 (60.4%) | Caucasian, 454 (94.4%) | Non-alcoholic fatty liver disease, 11 (2.3%) Cirrhosis, 23 (4.8%) HbsAg+ coinfection, 35 (7.3%) HCV PCR+ coinfection, 100 (20.7%) | Illicit drug use, 265 (55.0%)  Ongoing alcohol use, 109 (22.6%) |
| CDC 2018 [22] | 2012–2016 | Surveillance report | NNDSS | Incidence (2016): 0–9 years: 0.1 per 100,000 20–29 years: 0.9 per 100,000 30–39 years: 0.9 per 100,000 | Incidence (2016): Males: 0.7 per 100,000 Females: 0.6 per 100,000 | Incidence (2016): Asian/Pacific Islander: 1.5 per 100,000 | NR | 2016: Injection-drug use: 19 (0.9%) MSM: 8 (0.4%)  International travel: 34 (1.7%)  Note: majority of data is missing for risk factors, leading to low %s |
| CDC 2022* [24] | 2016 | CDC outbreak investigation | CDC/FDA | NR | NR | NR | NR | NR |
| CDC 2019 [21] | 2013–2017 | Surveillance report | NNDSS | 2016:  0–9 years: 47 (2.3%)  10–19 years: 131 (6.5%)  20–29 years: 392 (19.5%)  30–39 years: 391 (19.5%)  40–49 years: 333 (16.6%)  50–59 years: 297 (14.8%)  60+ years: 409 (20.4%)  2017:  0–9 years: 40 (1.2%)  10–19 years: 86 (2.6%)  20–29 years: 659 (19.6%)  30–39 years: 893 (26.5%)  40–49 years: 621 (18.4%)  50–59 years: 554 (16.5%)  60+ years: 509 (15.1%) | 2016: 1,107 (55.2%)  2017: 2,209 (65.6%) | 2016:  American Indian/Alaska Native: 3 (0.2%) Asian/Pacific Islander: 299 (21.5%) Black NH: 137 (9.9%) White NH: 865 (62.2%) Hispanic: 293 (21.1%)  2017: American Indian/Alaska Native: 13 (0.4%) Asian/Pacific Islander: 124 (3.7%) Black NH: 303 (9.0%) White NH: 1,979 (58.8%) Hispanic: 471 (14.0%) | NR | 2017: Injection drug use, 349 (10.4%) MSM, 95 (2.8%)  Sexual/household contact of HepA patient, 116 (3.4%) Other contact of HepA patient, 70 (2.1%) Contact of child/employee in a daycare center, 25 (0.7%) Child/employee in a daycare center, 20 (0.6%) International travel, 18 (0.5%)  Note: majority of risk factor data is missing, leading to low %s |
| CDC 2020 [20] | 2003–2018 | Surveillance report | CDC/NNDSS | 2018: 0–9 years: 54 (43.3%) 10–19 years: 231 (1.85%) 20–29 years: 2,763 (22.15%) 30–39 years: 4,268 (34.2%) 40–49 years: 2,658 (21.3%) 50–59 years: 1,509 (12.1%) 60+ years: 987 (7.9%) | 2018: 7,497 (60.1%) | 2018: American Indian/Alaskan Native: 15 (0.12%) Asian/Pacific Islander: 104 (0.83%) Black NH: 508 (4.1%) White NH: 8,670 (69.5%) Hispanic: 413 (3.3%) | NR | Injection drug use: 4,247/8,471 (50%) MSM: 194 (2.3%)  Sexual contact: 299 (3.4%) Household contact (non-sexual): 260 (3.1%) Other contact: 312 (3.7%) International travel: 114 (1.3%)  Injection drug use; sexual, household, or other contact; MSM; travel to hepatitis A-endemic region: 5,026 (40%) |
| CDC 2021 [19] | 2012–2019 | Surveillance report | NNDSS | 2019: 0–9 years: 127 (0.67%) 10–19 years: 231 (1.2%) 20–29 years: 3,582 (19%) 30–39 years: 6,400 (34%) 40–49: 4,177 (22.2%) 50–59 years: 2,635 (14%) 60+ years: 1,691 (9%) | 2019: 11,824 (62.7%) | 2019:  American Indian/Alaska Native: 60 (0.3%)  Asian/Pacific Islander: 139 (0.7%)  Black NH: 1,072 (5.7%)  White NH: 13,709 (72.7%)  Hispanic: 916 (4.9%) | NR | 2019:  Injection drug use, 5,017 (NR)  MSM, 201 (NR)  Sexual contact, 693 (NR)  Household contact (non-sexual), 563 (NR)  Other contact, 773 (NR)  International travel, 159 (NR) |
| CDC 2022* [23] | March 2022–August 2022 | CDC outbreak investigation | FDA | Median (range): 57 (9–73) years | 6 (32%) | NR | NR | NR |
| CDC 2022 [2] | 2016–2022 | CDC outbreak investigation | CDC Division of Viral Hepatitis | NR | NR | NR | NR | NR |
| Croker 2018 [25] | 2017 | Outbreak investigation | Los Angeles Country Department of Public Health | Range: 25–69 years | 3 (75%) | NR | NR | NR |
| Dankwa 2021 [26] | September 2017–June 2019 | Epidemic model | Surveillance data by LMPHW | 10–19 years: 5 (1.0%)  20–29 years: 95 (19.0%)  30–39 years: 185 (36.9%)  40–49 years: 120 (24.0%)  50–59 years: 65 (13.0%)  60–69 years: 23 (4.5%)  70+ years: 8 (1.6%) | 332 (66.3%) | NR | NR | Homeless, 128 (25.5%)  Illicit drug use IV, 276 (55.1%)  Illicit drug use non-IV, 189 (37.7%)  Persons experiencing homelessness or who use drugs, 385 (76.8%) |
| Foster 2018 [27] | 2007–2017 *Data reflects 2012–2017 | Retrospective cohort study | CDC/ NNDSS | NR | NR | NR | NR | NR |
| Foster 2018 [28] | 2017 | Report | CDC/ NNDSS | Median (range):  CA: 42 (5–87) years KY: 36 (1–84) years MI: 41 (<1–90) years UT: 38 (22–83) years | Total: 1,019 (67%) CA: 471 (69%) KY: 39 (66%) MI: 412 (65%) UT: 97 (66%) | NR | Total HepB coinfection: 42 (3%) HepC coinfection: 341 (22%)  CA HepB coinfection: 10 (1%) HepC coinfection: 116 (17%)  KY HepB coinfection: 4 (7%)  HepC coinfection: 29 (49%)  MI HepB coinfection: 16 (3%) HepC coinfection: 165 (26%)  UT HepB coinfection: 12 (8%) HepC coinfection: 31 (21%) | Total Homelessness and drug use: 395 (26%) Homelessness only: 78 (5%) Homelessness, drug use unknown: 51 (3%) Drug use only: 265 (17%) Drug use, homelessness unknown: 77 (5%) Neither homelessness nor drug use: 504 (33%) MSM: 81 (5%) Unknown: 114 (8%)  CA Homelessness and drug use: 247 (36%) Homelessness only: 65 (10%) Homelessness, drug use unknown: 43 (6%) Drug use only: 67 (10%) Drug use, homelessness unknown: 11 (2%) Neither homelessness nor drug use: 190 (28%) MSM: 18 (3%) Unknown: 59 (9%)  KY Homelessness and drug use: 27 (46%) Homelessness only: 3 (5%) Homelessness, drug use unknown: 2 (3%) Drug use only: 11 (19%) Drug use, homelessness unknown: 1 (2%) Neither homelessness nor drug use: 13 (22%) MSM: 4 (7%) Unknown: 2 (3%)  MI Homelessness and drug use: 64 (10%) Homelessness only: 7 (1%) Homelessness, drug use unknown: 2 (0.3%) Drug use only: 165 (26%) Drug use, homelessness unknown: 58 (9%) Neither homelessness nor drug use: 286 (45%) MSM: 61 (10%) Unknown: 27 (4%)  UT Homelessness and drug use: 75 (51%) Homelessness only: 5 (3%) Homelessness, drug use unknown: 5 (3%) Drug use only: 28 (19%) Drug use, homelessness unknown: 7 (5%) Neither homelessness nor drug use: 15 (10%) MSM: 1 (0.7%) Unknown: 13 (9%) |
| Foster 2021 [29] | January 1, 2017–October 31, 2018 | Report | CDC | Median (range): 32 (19–75) years | 260 (100%) | NR | HepB infection: 5/212 (2%) HepC infection: 2/212 (1%) HIV infection: 26/72 (36%) | MSM: 260 (100%) Injection or non-injection drug use during incubation period: 59/244 (24%)  International travel during incubation period: 54/253 (21%) |
| Haddix 2020 [30] | October 2018–April 2019 | Report | Cases reported to Los Angeles County Department of Public Health | Outbreak case definition: 18–34 years: 1 (14.3%) 35–44 years: 3 (42.9%) 55–64 years: 1 (14.3%) ≥75 years: 2 (28.6%)  Surveillance case definition: 18–34 years: 3 (30%) 35–44 years: 4 (40%) 45–54 years: 1 (10%) 55–64 years: 1 (10%) 65–74 years: 1 (10%) | NR | NR | NR | Outbreak definition: Homeless: 3 (42.8%) Illegal drug use: 3 (42.8%)  National surveillance case definition: Homeless: 4 (40%) Illegal drug use: 5 (50%) |
| Hagan 2022 [31] | 2016–2020 | Cross-sectional survey | Records from 25 health departments (reported via cross-sectional survey) | NR | NR | NR | NR | Among total population: Recently incarcerated: 2,093 (11.4%)  Among recently incarcerated: Drug use: 1,513/1,896 (79.8%) Homelessness: 560/1,700 (32.9%) MSM: 27/1,092 (2.5%)  HepA risk factors: 1,513/1,896 (79.8%) |
| Hofmeister 2020 [14] | 2017 | Retrospective cohort | Healthcare Cost and Utilization Project NIS | NR | NR | NR | NR | NR |
| Hofmeister 2021 [32] | July 1, 2016–June 10, 2019 | Matched case-control | State health department and hospital medical records | Cases Mean (SE): 53.6 (1.4) years  Median (range): 55.0 (24.0–87.0) years  20–29 years: 6 (5.5%) 30–39 years: 11 (10.0%) 40–49 years: 28 (25.5%) 50–69 years: 47 (42.7%) ≥70 years: 18 (16.4%)  Controls Mean (SE): 51.9 (0.7) years  Median (range): 53.0 (21.0–90.0) years  20–29 years: 31 (5.1%) 30–39 years: 51 (12.3%) 40–49 years: 104 (25.1%) 50–69 years: 189 (45.7%) ≥70 years: 49 (11.8%) | Cases: 75 (68.2%) Controls: 264 (63.8%) | Cases Caucasian/NH: 85 (96.6%) African American/NH: 3 (3.4%)  Hispanic: 0 (0.0%) Other: 0 (0.0%)  Controls Caucasian/NH: 195 (84.8%) African American/NH: 33 (14.3%)  Hispanic: 2 (0.9%) Other: 2 (0.9%) | Cases History of HepB: 22 (1.8%) History of HepC: 49 (46.2%) Pre-existing Liver Disease: 60 (81.1%) Diabetes: 41 (42.7%) Pregnancy: 0 (0.0%) Immunosuppression: 7 (8.1%)  Cardiovascular disease: 66 (69.5%)  Controls History of HepB: 36 (10.8%) History of HepC: 142 (39.1%) Pre-existing Liver Disease: 55 (49.1%) Diabetes: 60 (22.9%) Pregnancy: 3 (3.3%) Immunosuppression: 12 (5.9%) Cardiovascular disease: 125 (52.7%) | Cases Drug Use: 47 (52.2%) Injection Drug Use: 31 (39.2%) Non-injection Drug Use: 22 (33.8%) Homelessness, Unstable Housing, or Transient Living: 11 (11.2%) MSM: 3 (15.8%) Incarcerated: 3 (7.5%) International travel: 0 (0.0%) Epidemiologically linked: 14 (51.9%)  Controls Drug Use: 188 (55.1%) Injection Drug Use: 122 (39.4%)  Non-injection Drug Use: 109 (41.1%) Homelessness, Unstable Housing, or Transient Living: 38 (10.8%) MSM: 14 (12.2%) Incarcerated: 34 (16.7%) International travel: 0 (0.0%) Epidemiologically linked: 66 (50.4%) |
| Hofmeister 2021 [33] | July 1, 2016–June 10, 2019 | Retrospective, cross-sectional observational | KY Department for Public Health, the MI Department of Health and Human Services, the WV Bureau for Public Health | Overall ≤19 years: 16 (2.0%) 20–29 years: 168 (20.6%) 30–39 years: 292 (35.7%) 40-49 years: 188 (23.0%) 50–59 years: 100 (12.2%) 60-69 years: 36 (4.4%) ≥70 years: 17 (2.1%)  KY: ≤19 years: 14 (3.0%) 20-29 years: 99 (21.0%) 30–39 years: 172 (36.4%) 40–49 years: 115 (24.4%) 50–59 years: 52 (11.0%) 60–69 years: 13 (2.8%) ≥70 years: 7 (1.5%)  MI: ≤19 years: 0 (0.0%) 20–29 years: 15 (16.3%) 30–39 years: 31 (33.7%) 40–49 years: 14 (15.2%) 50–59 years: 18 (19.6%) 60–69 years: 10 (10.9%) ≥70 years: 4 (4.3%)  WV: ≤19 years: 2 (0.8%) 20–29 years: 54 (21.3%) 30–39 years: 89 (35.2%) 40–49 years: 59 (23.3%) 50–59 years: 30 (11.9%) 60–69 years: 13 (5.1%) ≥70 years: 6 (2.4%) | Overall: 511 (62.5%) KY: 297 (62.9%) MI: 63 (68.5%) WV: 151 (59.7%) | Overall White/NH: 337/368 (91.6%) African American/NH: 26/368 (7.1%) Other (Hispanic ethnicity or white and American Indian/Alaska Native): 5/368 (1.4%)  KY  White/NH: 155 (97.5%) African American/NH: 4 (2.5%) Other (Hispanic ethnicity or white and American Indian/Alaska Native): 0 (0%)  MI White/NH: 69 (76.7%) African American/NH: 16 (17.8%) Other (Hispanic ethnicity or white and American Indian/Alaska Native): 5 (5.6%)  WV White/NH: 113 (95.0%) African American/NH: 6 (5.0%) Other (Hispanic ethnicity or white and American Indian/Alaska Native): 0 (0%) | Overall History of HepB: 83/662 (12.5%) History of HepC: 370/723 (51.2%) Pre-existing Liver Disease: 83/186 (44.6%) Diabetes: 61/448 (13.6%) Pregnancy: 11/140 (7.9%) Immunosuppression: 8/347 (2.1%) Cardiovascular disease: 138/425 (32.5%)  KY History of HepB: 58 (15.3%) History of HepC: 226 (53.7%) Pre-existing Liver Disease: 46 (40.7%) Diabetes: 29 (14.0%) Pregnancy: 10 (13.3%) Immunosuppression: 6 (3.6%) Cardiovascular disease: 63 (30.4%)  MI History of HepB: 6 (7.2%) History of HepC: 22 (26.2%) Pre-existing Liver Disease: 9 (25.7%) Diabetes: 7 (8.2%) Pregnancy: 0 (0.0%) Immunosuppression: 1 (2.0%) Cardiovascular disease: 22 (35.5%)  WV History of HepB: 19 (9.5%) History of HepC: 122 (56.0%) Pre-existing Liver Disease: 28 (73.7%) Diabetes: 25 (16.0%) Pregnancy: 1 (2.4%) Immunosuppression: 1 (0.6%) Cardiovascular disease: 53 (34.0%) | Overall Drug Use: 459/627 (73.2%) Injection Drug Use: 329/557 (59.1%) Non-injection Drug Use: 267/450 (59.3%) Homelessness, Unstable Housing, or Transient Living: 92/656 (14.0%) MSM: 15/149 (10.1%) Incarcerated: 94/317 (29.7%) International Travel: 1/446 (0.2%) Epidemiologically Linked: 141/229 (61.6%)  KY Drug Use: 260 (78.3%) Injection Drug Use: 189 (68.7%) Non-injection Drug Use: 116 (61.7%) Homelessness, Unstable Housing, or Transient Living: 50 (11.7%) MSM: 4 (4.3%) Incarcerated: 68 (46.3%) International Travel: 0 (0.0%) Epidemiologically Linked: 72 (60.5%)  MI Drug Use: 47 (56.0%) Injection Drug Use: 27 (33.3%) Non-injection Drug Use: 37 (48.1%) Homelessness, Unstable Housing, or Transient Living: 10 (15.4%) MSM: 11 (22.4%) Incarcerated: 10 (12.7%) International Travel: 1 (1.2%) Epidemiologically Linked: 14 (34.1%)  WV Drug Use: 152 (72.0%) Injection Drug Use: 113 (56.2%) Non-injection Drug Use: 114 (61.6%) Homelessness, Unstable Housing, or Transient Living: 32 (19.8%) MSM: 0 (0.0%) Incarcerated: 16 (17.6%) International Travel: 0 (0.0%) Epidemiologically Linked: 55 (79.7%) |
| Hosseini 2018 [34] | 2016–2017 | Epidemiologic and morphologic review | San Diego Public Health Department | San Diego County: median (range): 43 (5–87) years  UCSD: median (range): 45 (23–100) years | San Diego County: NR (69%) USCD: NR (71%) | NR | UCSD Concurrent HepB: NR (5.5%) Concurrent HepC: NR (18.5%) | NR |
| Ismail 2020 [35] | 2017–2018 | Retrospective modelling study | NR | NR | NR | NR | NR | NR |
| Jiang 2019 [36] | November 1, 2016–October 10, 2017 | Retrospective case series | EMR of all patients hospitalized at the University of California San Diego | Mean (SD): 46 (11.9) years | 75 (70.8%) | Caucasian: 72 (67.9%) African American: 17 (16%) Hispanic: 17 (16%) | History of cirrhosis: 3 (2.8%) Alcohol use disorder history: 46 (44.7%) HepB positive: 3 (2.8%) HepC positive: 7 (6.9%) HIV: 1 | Homeless: 73 (68.9%) SUD: 59% |
| Kaigh 2020 [37] | July 16–October 8, 2019 | Retrospective review | EHR data | NR | NR | NR | NR | NR |
| Kang 2020 [38] | November 2016–October 2018 | Retrospective review | University of California San Diego Health, San Diego County Public Health | NR | NR | NR | NR | Among those receiving vaccinations (N=10,324): Homelessness: 1,385 (13%) Illicit drug use: 2,477 (24%) Alcohol abuse: 970 (9%) HepC infection: 104 (1%) Hepatitis B infection: 0 (0%) HIV infection: 585 (6%) Cirrhosis: 538 (5%) |
| Kreshak 2018 [39] | November 1, 2016–February 28, 2018 | Retrospective case series | EHR of tertiary care university hospital system’s two EDs (one urban and one suburban) | Mean: 45.1 years | 91 (68.4%) | NH: 113 (85.0%) | NR | Homeless: 86 (64.7%) Ethanol use: 70 (52.6%) Injection or illicit drug use: 53 (39.8%) |
| Lee 2020 [40] | 2011–2017 | Retrospective database review | NIS | Deceased:71.1 year (measure NR) Survivors: 59.9 years (measure NR) | Deceased: NR (48.2%) Survivors: NR (55.0%) | Deceased White: NR (74.6%) Black: NR (12.3%) Hispanic: NR (5.26%) Asian or Pacific Islander: NR (5.26%) Other: NR (2.63%)  Survivors White: NR (66.1%) Black: NR (19.2%) Hispanic: NR (9.73%) Asian or Pacific Islander: NR (1.70%) Other: NR  (3.35%) | NR | NR |
| Oller 2021 [41] | October 4, 2018–July 1, 2019 | Retrospective cohort | University of Kentucky | Mean (SE) Non-Bup/NX users = 41.3 (2.5) years Bup/NX users = 35.1 (2.1) years | Total, 18 (58%) Non-Bup/NX user, 9 (64%) Bup/NX user, 9 (53%) | Total: White, 29 (94%) Black, 1 (3%) Did not state, 1 (3%)  Non-Bup/NX users White, 13 (93%) Black, 0 (0%) Did not state, 1 (7%)  Bup/NX user White, 16 (94%) Black, 1 (6%) Did not state, 0 (0%) | Total HBV test surface antigen positive, 6 (19%) HBV test surface antibody positive, 11 (35%) HCV antibody positive, 24 (77%) HCV VL positive, 7 (23%) HIV, 0 (0%)  Non-Bup/NX user HBV test surface antigen positive, 4 (29%) HBV test surface antibody positive, 5 (36%) HCV antibody positive, 10 (71%) HCV VL positive, 3 (21%) HIV, 0 (0%)  Bup/NX user HBV surface antigen positive, 2 (12%) HBV surface antibody positive, 6 (35%) HCV antibody positive, 14 (82%) HCV VL positive, 4 (23%) HIV, 0 (0%) | Total  Past/present injection drug use, 28 (90%)  SUD diagnosis, 31 (100%) OUD only, 14 (45%) Stimulant use disorder only, 2 (6%) Alcohol use disorder only, 1 (3%) Multiple SUD, 14 (45%)  Non-Bup/NX user  SUD diagnosis, 14 (100%) OUD only, 6 (43%) Stimulant use disorder only, 2 (14%) Alcohol use disorder only, 1 (7%) Multiple SUD, 5 (36%)  Bup/NX user  OUD only, 8 (47%) Stimulant use disorder only, 0 (0%) Alcohol use disorder only, 0 (0%) Multiple SUD, 9 (53%) |
| Peak 2020 [42] | November 1, 2016–May 23, 2018 | Test negative case-control study | COSD | Median (IQR): 43 (34–52) years | 400 (67.9%) | NR | HBV coinfection, 25 (5.1%) HCV coinfection, 83 (17.5%)  Underlying factors: 19/20 patients who died of HepA-associated causes (95%)  Relapsing HAV: 2/20 patients who died of HepA-associated causes (10%) | Homeless and illicit drug use, 200 (34.0%) Homeless only, 91 (15.4%) Illicit drug use only, 77 (13.1%) Neither, 167 (28.4%) Unknown, 54 (9.2%)  ACIP indications: Any, 324 (54.6%) Illicit drug use, 277 (47.0%) Coinfection with HBV or HCV, 101 (20.1%) International travel, 23 (5.3%) MSM, 14 (3.5%) Aged <18 years, 2 (0.3%) |
| Reichenbach 2021 [43] | May 2017 –December 2019 | Retrospective chart review | Temple University Hospital | Age (SEM) years: 46.12 (1.06) 20–29 years, 21 (10.24%) 30–39 years, 68 (33.17%) 40–49 years, 40 (19.51%) 50–59 years, 30 (14.63%) 60–69 years, 26 (12.68%) 70–79 years, 15 (7.32%) 80–89 years, 5 (2.44%) | 137 (66.8) | African American/Black, 43 (21.0%) Caucasian/white, 77 (37.6%) Hispanic, 66 (32.2%) Other/unknown, 19 (9.3%) | Cirrhosis on imaging, 6/156 (3.9%) | History of substance abuse, 85/172 (49.4%) Homelessness, 34/141 (19.4%) |
| Samala 2021 [44] | October 2015–April 2019 | Retrospective analysis | Indiana University Health (largest, most comprehensive healthcare system in state), Eskenazi Health (largest county health system in state) | Mean (SD): 40.95 (14.49) years | 164 (62) | Caucasian, 249 (94%) Black, 6 (2%) Asian, 1 (0.4%) Native America, 3 (1.0%)  Hispanic, 1 (0.4%) | Underlying CLD, 120 (45%) HepC infection, 85 (71%) HepB injection, 3 (2%) HepB and C coinfection, 3 (2%) Non-alcoholic fatty liver disease, 12 (10%) Alcoholic liver disease, 7 (6%) Autoimmune hepatitis, 2 (2%) HepC and alcohol, 1 (1%) Cryptogenic cirrhosis, 6 (5%) Hepatocellular cancer, 1(1%) Underlying cirrhosis, 20 (8.0%) Hypertension, 54 (20%) Dyslipidemia, 24 (9%) Obesity, 60 (23%) Congestive heart failure, 7 (3%) Diabetes mellitus type 2, 21 (8.0%) Hypothyroidism, 12 (4.0%) HIV/AIDS, 3 (1.0%) Coronary artery disease, 9 (3%) Cancer, 6 (2.0%) Other, 11 (4.0%) Autoimmune disease, 1 (4%) Unknown, 7 (3%) | Mode of transmission:  Any drug use, 199 (74.0%)  Injection drug use, 147 (74.0%)  Methamphetamine, 108 (54.0%)  Heroin, 54 (27.0%)  Prescription opiates, 12 (6.0%)  Cocaine, 7 (3.0%)  History of incarceration, 28 (11.0%) Poor living conditions, 22 (8.0%) Sexual partner with HepA, 10 (4.0%) Travel to HepA endemic area, 4 (1.0%)  Food source, 41 (15.0%) Person-to-person, 29 (11.0%)  Unspecified/other, 8 (4.0%) |
| Viray 2019* [45] | June 11, 2016–October 22, 2016 | Outbreak investigation | Medical records and other information (report of government investigation, findings, and public health response) | Median (range): 40 (16–80) years | 172 (59%) | Asian, 195 (69%) Native Hawaiian/Pacific Islander, 31 (11%) White, 29 (10%) Black, 1 (0%) Two or more races, 26 (9%) | NR | NR |
| Wilson 2019 [46] | January 2018–August 2018 | Retrospective cohort | Kanawha-Charleston Health Department data (otherwise NR) | Median (range): 37 (14–77) years | 398 (60%) | NR | Past or current HCV infection: 314 (47%) Past or current HBV infection: 65 (10%) | Current or past illicit drug use: 540 (81%) Homeless or having a transient living situation: 100 (15%) |

* Outbreak(s) occurred solely via food-borne transmission.

^a^ Outbreak-associated cases were defined as HAV infections occurring in persons who 1) resided or spent time in the outbreak area during October 15, 2018–April 29, 2019 and 2) either had infections caused by the HAV genotype IB CA Cls A strain or were epidemiologically linked to a person infected with the outbreak strain.

^b^ National surveillance acute hepatitis A case definition in 2018: acute illness with discrete onset of symptoms consistent with acute viral hepatitis, jaundice or

elevated ALT or aspartate aminotransferase, and IgM antibody to hepatitis A virus (anti-HAV) positive.

Abbreviations: ACIP, Advisory Committee on Immunization Practices; AIDS, acquired immunodeficiency syndrome; ALT, alanine transaminase; Bup, buprenorphine; CA, California; CDC, Centers for Disease Control and Prevention; CLD, chronic liver disease; COSD, City of San Diego; ED, emergency department; EHR, electronic health record; EMR, Electronic Medical Record; FDA, Food and Drug Administration; HBV, hepatitis B virus; HbsAg, hepatitis B surface antigen; HCV, hepatitis C virus; HepA, Hepatitis A; Hep B, Hepatitis B; HepC, Hepatitis C; HIV, human immunodeficiency virus; IgM, immunoglobin M; IN, Indiana; IQR, interquartile range; IV, intravenous; KY, Kentucky; LMPHW, Louisville Metro Department of Public Health and Wellness; MDSS, Michigan Disease Surveillance System; MSM, men who have sex with men; NNDSS, National Notifiable Diseases Surveillance System; NH, non-Hispanic; NIS, network and information systems; NR, not reported; NX, naxalone; OUD, opioid use disorder; PCR, polymerase chain reaction; SD, standard deviation; SEM, standard error of the mean; SE, standard error; SUD, substance use disorder; TN, Tennessee; UK, United Kingdom; USCD, University of California, San Diego; UT, Utah; VL, viral load; WI, Wisconsin; WV, West Virginia.

**Supplementary Table 5.** Outbreak management considerations of studies included in the TLR

| **Reference** | - **Location** - **Study population**   **(N cases)** | **Outbreak management considerations** | | | |
| --- | --- | --- | --- | --- | --- |
|  |  | **Outbreak management tools** | **Key successes** | **Key challenges** | **Key learnings** |
| Baloch 2021 [76] | - IL, WI - Patients from rural and urban populations who did not have diagnosis of HepA and received HepA vaccine (N=NR) | Vaccination | NR | NR | - Notable disparities in vaccination among groups |
| Brouwer 2020 [17] | - MI - All positive HepA cases reported to the MDSS (N=910) | Vaccination | - High HAV aversion due to vaccination outside of Southeast MI | - Low HAV aversion due to vaccination in Southeast MI - Insufficient targeting of at-risk individuals and logistic and resource challenges | - Increased vaccination coverage earlier in the outbreak (ie, larger coverage [better targeting or more doses administered] and earlier vaccination timing) would have a higher impact on HAV infection - Proactive control strategies are more effective |
| Buechler 2020 [65] | - Detroit, MI - Adults living in Detroit, MI who lacked permanent housing (44 total participants [HepA status NR]; 5 had contact with someone known or suspected to have HAV in past year) | Vaccination; Hygiene practices | NR | - Difficulty accessing vaccines - Vaccine hesitancy/refusal due to fear or distrust - Significant barriers to hygiene practices, including lack of access to showers and laundry facilities - Challenges of contact-tracing | - Significant barriers in hygiene practices and vaccination that should be addressed to reduce HAV infections |
| Butt 2020 [18] | - Charleston, WV - Patients with HepA who were hospitalized (N=482) | Testing of illicit drugs, vaccination, and educational efforts | NR | NR | - Potential link between HAV and IV methamphetamine use, indicating possible new route of transmission, requiring new intervention tools - Given potential link between HAV and IV methamphetamine use, interventions should be targeted towards vulnerable populations |
| Campos-Outcalt 2018 [69] | - National - Cases of HepA reported to CDC from CA, MI and UT - 1995 (national): N=271,000; 2015 (national): N=2,800; 2017 (in NYC): N=51; 2017 (in CA): N=672; 2017 (in southeastern MI): 555; 2017 (in UT): N=91 | Vaccination | - Reduction in HAV cases due to vaccination | - Despite lower rates of HAV, people with comorbidities remain susceptible - Vaccination supply strained by large outbreaks, so physicians need to be aware of local availability and regulations | NR |
| Castillo 2018 [59] | - San Diego, CA - HepA patients that were treated in the ED and either discharged home or transferred (N=582 HepA cases; 1,480 ED visits in study) | Automated computerized alert, or BPA, embedded within the EHR (Epic Systems, Madison, WI) to prompt providers to order and administer the HAV vaccine for patients identified as homeless in the EHR | - Higher rates of vaccination among targeted homeless population | NR | - Informatics tools are successful in increasing vaccination efforts in hard-to-reach populations |
| CDC 2020 [58] | - MI - HepA cases from Michigan reported to CDC (N=907) | Vaccination; health communication materials | - Decline in number of new cases since December 2017 - Partnerships allowed for expanded reach to at-risk populations | NR | NR |
| County of San Diego 2018 [55] | - San Diego County, CA - HepA cases reported by the County of San Diego (N=582) | Vaccination; Sanitation, Education; Outbreak Emergency Management | - Increase in public awareness about outbreak - Reverse increasing trend of HAV infections - Early detection and urgent, coordinated response | - Homelessness/drug use | - Need for improved collaboration between partners - Need for formal incident management for outbreaks - Need for policy group that includes leadership/representation from jurisdictions |
| Dankwa 2021 [26] | - Louisville, KY - HepA cases captured by surveillance conducted by the LMPHW (N=501) | Vaccination | - HAV case prevention - Vaccination program mitigated spread to other counties | - Time/data required before implementation | - Use readily available data for more timely intervention implementation |
| Duncan 2018 [52] | - Clinics in San Diego and El Cajon, CA - Adults receiving HepA vaccines at FHCSD clinics (N=19) | Vaccination | - Partnerships between organizations expanded outreach - Improvement in health systems for greater identification of those eligible for vaccination and tracking vaccination - Increased healthcare staff's ability to administer vaccine | - Vaccines distrusted among homeless/homeless do not consider themselves at risk for HAV - Media coverage may reach wrong audience/those who are not at high risk for HAV may monopolize resources better spent on high-risk populations | - Effective outbreak management includes quick action, mobilization of multiple departments, and leveraged relationships - Executive leadership support provides direction and coordination - Relationships with other service providers allow for network of support for at-risk populations and for rapid community outreach |
| Felner 2020 [67] | - San Diego, CA - TAY (18–24 years) experiencing housing instability or homelessness (N=NR; TAY, N=7) | Vaccination, handwashing stations, sanitation of sidewalks and public spaces, and distribution of personal hygiene kits | NR | NR | - TAY perceived public health efforts to be insufficient - Lack of handwashing stations and public bathrooms - Stigma against homeless - Exclusivity of resources - Lack of coordination across support resources - Over-policing and criminalization - Racism |
| Fukuda 2020 [57] | - MA - HepA cases identified by MA integrated surveillance system (N=NR) | Policies that mandated contracted organizations to submit specimens for testing to the Massachusetts State Public Health Laboratory; co-test blood specimens for HIV, HCV, and syphilis; integrate HIV, viral hepatitis, and STI disease surveillance and case management in a single data system; and implement an integrated infectious disease drug assistance program | - Prompt identification of outbreaks - Agencies connected to appropriate areas of risk - Increased vaccination efforts | - External billing agent - Extensive negotiation - Extensive training required - Lack of communication on insurance - Difficult to confirm chronic HCV potential - High proportion of HIV diagnoses | NR |
| Haddix 2020 [30] | - Los Angeles County, CA - Outbreak-associated HepA cases identified by the Los Angeles County Department of Health (Outbreak case definition^a^, N=7; Surveillance case definition^b^, N=10) | Vaccination, environmental health outreach, health care provider alerts about the increased occurrence of HepA | - Improved surveillance and clinics - Increased HepA vaccination - Education to restaurant workers | - Increased resource requirement due to challenging HAV genotyping | NR |
| Hagan 2022 [31] | - AZ, AR, CA, (San Diego), CO, DE, FL, GA, IL, LA, MA, MI, MN, MS, NV (Southern NV Health District), NH, NM, NY (excluding NYC), NC, OH, PA (Philadelphia), TN, UT, VT, VA, WA - HepA cases identified by state and local health departments (Overall, N=18,327; Recently incarcerated, N=2,093) | Vaccination in correctional and detention facilities | NR | - HAV transmission occurring within correctional and detention facilities (limited information on this transmission environment) - Correctional and detention facility populations tend to have higher rates of risk factors than the general population and may have more difficulty seeking prevention services when not incarcerated - Correctional and detention facilities do not have resources to undertake vaccination campaign; strong partnerships with public health operations needed | - Persons with recent history of incarceration report high levels of drug use and should be included in outbreak management activities - Given HAV transmission occurring in correctional/detention facilities, vaccination should be targeted in jails (where most individuals are held) to prevent spread and contribute to outbreak prevention |
| Hu 2020 [70] | - Global - HepA cases associated with foodborne outbreaks (Viray et al. 2018, N=292; US CDC 2016, N=143; Collier et al. 2014, N=165) | Vaccination, contacting individuals directly, suspending contaminated food | - Collaboration contributed to prompt surveillance - Contacting individuals directly halted outbreaks - Suspending contaminated food halted outbreaks | - Challenges with vaccine supply | NR |
| Kaigh 2020 [37] | - Philadelphia, PA - All ED visits that were evaluated for HepA before and after a vaccination program (Before program initiation, N=73; After program initiation, N=38) | Vaccination program, BPA | - Large number of vaccinations - Drop in HepA cases - Collaboration between stakeholders - Other urban EDs using this model | - Population at risk (homeless) had limited access to primary care, so innovate approach needed | NR |
| Kang 2020 [38] | - San Diego County, CA - Confirmed and probable outbreak-associated cases of HepA in San Diego County (San Diego County, N=592; UCSDH, N=144) | Vaccinate, sanitize, educate | - Increased HepA vaccination | - Outbreak management and prevention | NR |
| Kronen 2022 [74] | - Boston, MA - Patients with cirrhosis in an inpatient setting (Cirrhosis cases, N=381; pre-intervention, N=187; post-intervention, N=194) | Step 1: checklist for hepatitis serologies and vaccination; Step 2: brief educational session for residents promoting hepatitis vaccination in patients with cirrhosis | - Improved vaccination rates | - No difference in serology testing after intervention | NR |
| Kushel 2018 [75] | - CA - People experiencing homelessness during HepA outbreaks (N=NR) | Vaccination, education, and provide portable hygiene facilities in areas where homeless individuals congregate | NR | - Continued homelessness could impact intervention success | - Important to treat symptoms (HepA/vaccination) as well as address root causes |
| Luder 2018 [72] | - OH, IN, KY - Patients filling at least 1 maintenance medication, with focus on high-risk patients (N=NR) | Appointment-based model: Patients made an appointment to pick up synchronized prescriptions, and pharmacists assessed vaccination histories and administered vaccinations during the appointment. Pharmacists used state-wide IIS to determine vaccination histories and document administered vaccines | - Increase in HepA vaccinations - Pharmacists and patients satisfied with appointment-based model | - Incomplete data in the IIS | - Usefulness of appointment-based model - Improvement in IIS data collection needed |
| McCosker 2022 [73] | - Australia, UK, US - People experiencing homelessness (N=NR) | Vaccination (campaign and clinic) and education directed at homeless persons, automated computerized alert in the ED | - In most vaccination strategies, vaccines were free | - Nurses and other staff may have negative view towards homeless; need training to improve attitudes - Effectiveness of education, reminders, and incentives is unclear and depends on strategy’s budget - Vaccination strategies may be paired with co-interventions, but limited funding, staff, and resources impede ability to effectively deliver   If vaccines are not free, cost is barrier to uptake | - Multiple options for ‘non-traditional’ vaccine delivery locations; should target areas convenient for target population - Accelerated vaccination schedules - Active recall (contacting people by telephone and locating them through other services and facilities) is a vital component of vaccination strategies - Vaccination of volunteers and staff is vital - Stakeholder collaboration key enabler for success |
| Montgomery 2022 [66] | - National - Adults working at the SSP for at least months (N=NR) | SSPs | NR | - Lack of funding and resources for SSPs - Lack of licensed staff for SSPs prohibits expansion of vaccination services - Lack of client demand for vaccinations | - HepA vaccines are included in SSPs: Among the 46 SSPs with vaccinations, 35 (76%) offered HepA vaccination - Expanding number of providers to administer vaccines will improve vaccination access in SSPs - Partnerships with health departments and health systems would help with staffing issues |
| Montgomery 2021 [60] | - National - People who use drugs and people experiencing homelessness during HepA outbreaks (N=NR) | Population engagement, vaccination, vaccination tracking | - Overall reduction in barriers to vaccine uptake - Improvement in vaccine acceptance and reduction in concerns about vaccine safety - Improved vaccine delivery methods | - Access, distrust, and stigma inhibit vaccine uptake - Lack of access to IIS to track outbreaks | - Population engagement and collaboration pivotal to success |
| NACCHO 2017 [61] | - CA, MI - HepA cases identified in San Diego, Detroit, and Los Angeles (San Diego [2016–2017], N=536; Detroit [August 2016–2017], N=457; Los Angeles County, N=14) | San Diego County: Vaccinate, Sanitize/Hygiene, Educate Detroit: Understanding the Risk, Prevention (Outreach, education, vaccination), Response (PEP, Protecting Key Personnel) Los Angeles County: Vaccination, Sanitation, Awareness Campaign | San Diego County   - Administration of vaccinations in a variety of settings - Provision of guidance during health inspection - Access to outdoor sanitation and hygiene supplies - Large reach during education events and initiatives  Detroit - Administration of vaccinations in a variety of settings  Los Angeles County - Administration/distribution of a larger number of vaccines | Detroit:   - Difficult to reach populations (geographic constraints) - High numbers of close contacts, partners lost to follow-up - Vaccine financing, shortage, delivery issues - Epidemiological support needed   Los Angeles County: - Various challenges to combatting - HepA in jail - Barriers to vaccine accessibility | Detroit   - Partnerships are critical |
| Oren 2020 [64] | - San Diego County, CA - Tweets limited to San Diego County (N=NR; 744 tweets analyzed) | Twitter communication about HepA outbreak (general Tweeting, no specific source) | - Misalignment on vaccination messaging between authorities and members of the public was minimal - Misinformation was limited | - Misalignment on hygiene and sanitation messaging between authorities and members of the public - Few tweets shared by government source suggesting lack of (meaningful) dialogue between agencies and public | - Dialogue between public and government is important to decrease public perception of insufficient action/unsatisfactory response on part of government - Misinformation may lead to propagation of incorrect information - Twitter may be a missed opportunity to contact high-risk populations (such as homeless) - Fast-paced spread of information allows propagation of anti-vaccination arguments, which could be addressed by public health professionals |
| Schwartz 2022 [54] | - NA/National - Facebook advertisement campaign for target audiences (MSM and FSW) (N=NR) | Facebook ad campaign: Five Facebook Ads were created that varied in images and text, but all included a link to the CDC website on HepA (https://www.cdc.gov/hepatitis/hav/) as the call to action. A set of advertisements was designed with targeted messaging set to display to specific target audiences on Facebook. There were 3 target audiences used for Facebook ads: (1) overall audience, (2) MSM, and (3) FSW | - Campaign was effective in reaching target audience | - Men are more difficult to reach than women in health promotion campaigns - Negative campaign feedback | - Consider other metrics to improve messaging via Facebook advertisements |
| Snyder 2019 [68] | - National - Health officials from 9 city or county health departments (N=11,628) | Infrastructure and sanitation, vaccination, outreach, and education | NR | - Difficulty in developing vaccination plans due to lack of access and guidance - Lack of trained staff to administer vaccine - Difficulty in vaccination tracking | - Important to build and maintain multisectoral partnerships - Need for innovative engagement strategies - Need for communication strategies with high-risk groups about HepA vaccines |
| Sunshine 2019 [71] | - San Diego, Santa Cruz, and Los Angeles counties, CA - HepA cases identified by the County of SDHHS (N= NR) | State of emergency declaration Vaccination efforts, education, vaccine supply management, and sanitation measures | - Decrease in outbreak cases | - Hard-to-reach population - Underlying conditions including mental illness, CLD, lack of sanitation, and lack of education - Constrained vaccine supply due to dramatically increased demand. | NR |
| Talbott 2019 [77] | - National - People identified by DPH who were targeted to receive HepA vaccine (N= NR) | Vaccination clinic set up at homeless or residential shelters; Vaccination clinic by DPH | NR | NR | NR |
| Ungar 2019 [56] | - KY, CA, TN, IN, MI - HepA cases identified in KY, CA, TN, IN, and MI (Kentucky [mid-Feb 2019], N=4,100; San Diego County [March 2017–January 2018], N=592; Tennessee [mid-Feb 2019], N=865; Indiana [mid-Feb 2019], N=1,203; Michigan, N=910) | San Diego County, CA: Vaccination TN: Vaccination IN: Deployment of “strike teams” to target at-risk populations MI: Education and vaccination outreach efforts; Emergency operations center to coordinate efforts | San Diego County, CA:   - Increase in number of people vaccinated - Decrease in new outbreak cases  TN - Early attention/preparation to outbreak   MI   - Targeting high-risk populations where they are - Recruitment of advocates | Kentucky:   - Lack of funding   TN:   - Lack of staff for vaccination administration | San Diego County, CA:   - Estimating how many people should be vaccinated/how much vaccine to give out - Quick acceleration of vaccine efforts to reduce risk of disease spreading |
| Viray 2019 [45] | - HI - Outbreak-associated HepA cases reported to HI DOH (N=292) | HDOH medical advisory to healthcare providers, CDC Epidemic Information Exchange alert, multiple attempts to contact all cases, case interviews, medical records of cases obtained for those who could not be contacted, online survey to estimate baseline commercial food consumption behaviors, review of shopper loyalty cards, laboratory testing/examinations of strain sequence and comparison to sequences in CDC’s databases and other foodborne HAV outbreaks, product investigation and trace-back, product laboratory testing (for suspected products), contact tracing, subset and GIS analyses to identify trends and clustering | - Routine childhood HAV vaccination may have led to minimal pediatric cases/minimal secondary spread - Use of alternate information (online survey) made it possible to obtain information needed to identify contaminant source - HAV genotyping/ sequencing distinguishes outbreak from other concurrent outbreaks and isolated cases | - Lower food safety standards in ex-US food imported into the US - Although FSMA approved in 2011, implementation still in progress and roll-out occurring over multiple years^a^; FSMA still does not address record keeping standards - Incomplete records/information from purchasers and distributors and delays in receiving information - Unclear where exactly contamination occurred in scallop harvest/processing process - Popularity of at-risk behaviors for HepA contamination (i.e., consumption of raw/undercooked seafood) - Baseline information needed to identify contaminant not always available - Few HAV genotype/sequencing samples submitted to CDC repository to provide additional information (helpful for outbreak identification and comparison against concurrent outbreaks and isolated cases) - Large period of potential exposure may cause misclassification of exposure status due to poor recollection - No formal, analytic study estimated the association between food exposure and case status | - Standardized (and well conducted) record keeping makes ID of contaminated products easier - Quick identification of contaminant leading to fast preventive response is important |
| Wooten 2019 [62] | - San Diego County, CA - HepA cases identified by SDHHS (N=592) | Vaccination campaign, address sanitation needs, address public health issues, partner with CDC and external organizations, implement ODS, homeless outreach | - Vaccination campaign targeted enhanced approach (public health centers; medical institutions) and new approach (jails during intake and to inmates; SUD treatment programs; homeless service providers; emergency departments; behavioral health system; law enforcement/paramedics; internal partners) - Vaccination field events: PODs, mobile van, foot teams, jails, tents, PEP. - As of 1/31/2019, 209,815 total vaccinations provided by the County, hospital partners, and community providers | - Surge capacity and staffing and training: obtaining surge staff (redirecting County employees, intermittent workers, temporary agency nurses, Medical Reserve Corps, healthcare system volunteers) and training (vaccine management and administration, safety for nurses working in jails, San Diego Immunization Registry, Outbreak Response Management System) - Disease-specific challenges - Population-specific challenges - Low vaccination rates among adults with CDC indications, isolation of discharged infectious homeless individuals, cross-over into other populations, communication to “worried well” | - Vaccination strategies: engage multiple agencies, utilize multiple strategies to reach at-risk persons, - Plan data collection elements in advance - Improve ability for homeless to maintain hygiene - Educate through multiple channels   Grand Jury Report Recommendations:   - Declare a local public health emergency much sooner when confronted with a similar outbreak in the future - Revise the county emergency operations plan to establish a command structure during a health emergency - Establish lines of authority to prevent misunderstandings regarding departmental responsibilities - Designate a project manager who can communicate effectively with city officials and medical personnel to take necessary actions quickly during a health emergency   CA State Auditor’s Report  Recommendations:   - Revise the county’s response plans to include specific and achievable objectives, schedules by which it expects to achieve these objectives, and resources necessary to achieve its goals and objectives within planned schedule - Finalize county’s draft policy that requires it to promptly convene policy groups with representatives from relevant local jurisdictions in a future outbreak - Enter in an agreement with the city of San Diego to clarify each entity’s roles and responsibilities over public health matters |
| Wooten 2019 [63] | - San Diego, CA - HepA cases identified in San Diego (N=NR [total cases]) | Vaccination, sanitation, education | - Robust, innovative, and individualized outbreak management strategies targeting those at highest risk - Identify and target risk factors early and use to guide outbreak response/management | - Homelessness and associated poor sanitation root cause of outbreak; more needs to be done to address homeless crisis | - Vaccination was most effective tool |
| Zellmer 2021 [78] | - Hennepin county, MN - People incarcerated in Hennepin County Jail Services (N=127 [total cases in the state]) | Vaccination in jails | - Vaccination rate increased after implementation of vaccination program - Single dose still confers some immunity and is short-term solution for outbreak Jail vaccinations become part of larger state-wide database, can be pulled up and subsequent vaccination care (e.g., second shot) provided at other facilities | - Rapid turnover of jail population makes vaccination difficult - Jailed persons expressed interest but ultimately did not want to be vaccinated - Incomplete HepA vaccination records (however, repeat HepA vaccination poses no significant risk) - Need for vaccine education - Needles possible triggers for patients with SUD | - Difficult to implement sustainable programs but modifications to existing protocols and minimal additional resources encourage sustainability - Hennepin County outbreak management shows role of jails in meeting health needs of marginalized populations and confer benefits to larger populations |

^a^ Outbreak-associated cases were defined as HAV infections occurring in persons who 1) resided or spent time in the outbreak area during October 15, 2018–April 29, 2019 and 2) either had infections caused by the HAV genotype IB CA Cls A strain or were epidemiologically linked to a person infected with the outbreak strain.

^b^ National surveillance acute hepatitis A case definition in 2018: acute illness with discrete onset of symptoms consistent with acute viral hepatitis, jaundice or elevated ALT or aspartate aminotransferase, and IgM antibody to hepatitis A virus (anti-HAV) positive.

Abbreviations: ALT, alanine transaminase; BPA, best practices alert; CA, California; CDC, Centers for Disease Control and Prevention; CLD, chronic liver disease; DOH, Department of Health; DPH, Delaware Division of Public Health; ED, emergency department; EHR, electronic health record; FHCSD, Family Health Centers of San Diego; FSMA, Food Safety Modernization Act; FSW, food service worker; GIS, geographic information system; HAV, hepatitis A virus; HCV, hepatitis C virus; HepA, hepatitis A; HI, Hawaii; HIV, human immunodeficiency virus; ID, infectious disease; IgM, immunoglobin M; IIS, Internet Information Services; IL, Illinois; IN, Indiana; IV, intravenous; KY, Kentucky; LMPHW, Louisville Metro Public Health and Wellness Department; MA, Massachusetts; MDSS, Michigan Disease Surveillance System; MI, Michigan; MN, Minnesota; MSM, men who have sex with men; NA, not applicable; NR, not reported; NYC, New York City; ODS, operational data store; OH, Ohio; PA, Pennsylvania; PCR, polymerase chain reaction; PEP, post-exposure prophylaxis; POD, point of dispensing; SD, standard deviation; SDHHS, San Diego Health & Human Services Agency; SSP, syringe services program; STI, sexually transmitted infection; SUD, substance use disorder; TAY, transitional aged youth; TN, Tennessee; UCSDH, University of California San Diego; UK, United Kingdom; US, United States; UT, Utah; WI, Wisconsin; WV, West Virginia.

**Supplementary Table 6.** Study design and patient population of studies included in the TLR

| **Reference** | **Study dates** | **Study design** | **Data source** | **Population primarily affected by outbreak** | | | | |
| --- | --- | --- | --- | --- | --- | --- | --- | --- |
|  |  |  |  | **Age (as reported)** | **Males**  **(N [%])** | **Race/ethnicity**  **(N [%])** | **Comorbidities**  **(N [%])** | **Risk factors**  **(N [%])** |
| Baloch 2021 [76] | NR | Retrospective cohort | NR | NR | NR | White, 88%  Non-Hispanic, 92.5% | NR | NR |
| Brouwer 2020 [17] | August 2016–December 2018 | Mathematical model | MDSS, MCIR | Median, 40 years | 594 (65%) | NR | History of HBV or HCV, 233 (26%) | Substance use, 427 (47%) Substance use (non-IV only), 172 (19%) Substance use (IV only), 71 (8%) Substance use (both), 159 (17%) Substance use (unknown), 25 (3%) Homeless/transient housing, 114 (13%) Institutional risk, 100 (11%) MSM, 79 (9%) None of the above, 311 (34%) |
| Buechler 2020 [65] | 2018 | Cross-sectional | Surveys | Mean, 50 years | 18 (41%) | Black, 39 (90%) | NR | NR |
| Butt 2020 [18] | March, 2017–November, 2018 | Retrospective chart review | Patient records at tertiary care center | Mean (SD): 42 (13.3) years | 291 (60.4%) | Caucasian, 454 (94.4%) | Non-alcoholic fatty liver disease, 11 (2.3%) Cirrhosis, 23 (4.8%) HbsAg+ coinfection, 35 (7.3%) HCV PCR+ coinfection, 100 (20.7%) | Illicit drug use, 265 (55.0%)  Methamphetamine use, 248 (51.5%) Heroin use, 74 (15.4%) Marijuana use, 139 (28.8%) Route of illicit drug use: IV drug use, 187 (38.8%) Oral drug use, 16 (3.3%) Both IV and oral drug use: 47 (9.8%) Neither IV nor oral drug use: 15 (3.1%)  Ongoing alcohol use, 109 (22.6%)  Non-alcoholic fatty liver disease, 11 (2.3%) Cirrhosis, 23 (4.8%)  Employment: 55 (11.4%) |
| Campos-Outcalt 2018* [69] | 1995–2018 | CDC report | CDC | NR | NR | NR | San Diego, CA:  Chronic HepC, 20% Chronic HepB, 5%  Southeastern MI and UT: Chronic HepC, 27.5% Chronic HepB, 13.5% Both, 9.9% | Prior to 2016 (national): contaminated food, infected food handlers, and other food service-related exposures  CA (2017): homeless and users of illicit drugs, whose risk of infection is compounded by exposure to fecally-contaminated environment  Southeastern MI and UT (2017): homelessness and illicit drug use  NYC (2017): MSM |
| Castillo 2018 [59] | 2016–2018 | Retrospective quasi-experimental | EHR data | NR | NR | NR | NR | NR |
| CDC 2020 [58] | 2016–2018 | Report/brief | NR | NR | NR | NR | NR | NR (specific numbers NR, but homeless, incarcerated, SUD, and MSM most likely to be affected) |
| County of San Diego 2018 [55] | 2017–2018 | Report | NR (possibly County health department) | NR | NR | NR | NR | NR |
| Dankwa 2021 [26] | 2017–2019 | Modelling study | LMPHW | 10–19 years: 5 (1.0%) 20–29 years: 95 (19.0%) 30–39 years: 185 (36.9%) 40–49 years: 120 (24.0%) 50–59 years: 65 (13.0%) 60–69 years: 23 (4.5%) 70+ years: 8 (1.6%) | 332 (66.3%) | NR | NR | Persons experiencing homelessness or who use drugs: 385 (76.8%) Homeless: 128 (25.5%) Illicit drug use, IV: 276 (55.1%) Illicit drug use, non-IV: 189 (37.7%) |
| Duncan 2018 [52] | March 10 2017–October 8, 2017 | Narrative review | NR | NR | NR | NR | NR | Homeless, 53% Illicit drug users, 68% |
| Felner 2020 [67] | 2016–2018 | Qualitative research study | Data collected by project team | Mean, 21.86 years (+/-1.57; range: 19–24) | NR (n=4 self-identified as members of LGBTQ community) | Person of color, 5 (71.4%) | NR | Homeless, 7 (100%) |
| Fukuda 2020 [57] | 2014–2018 | Case study | MAVEN | NR | NR | NR | NR | NR |
| Haddix 2020* [30] | October 2018–April 2019 | Report | Cases reported to Los Angeles County Department of Public Health | Outbreak case definition: 18–34 years: 1 (14.3%) 35–44 years: 3 (42.9%) 55–64 years: 1 (14.3%) ≥75 years: 2 (28.6%)  Surveillance case definition: 18–34 years: 3 (30%) 35–44 years: 4 (40%) 45–54 years: 1 (10%) 55–64 years: 1 (10%) 65–74 years: 1 (10%) | NR | NR | NR | Outbreak definition: Homeless: 3 (42.8%) Illegal drug use: 3 (42.8%)  National surveillance case definition: Homeless: 4 (40%) Illegal drug use: 5 (50%) |
| Hagan 2022 [31] | 2016–2020 | Cross-sectional survey | Records from 25 health departments (reported via cross-sectional survey) | NR | NR | NR | NR | Among total population: Recently incarcerated: 2,093 (11.4%)  Among recently incarcerated (N=2093) Drug use: 1,513/1,896 (79.8%) Homelessness: 560/1,700 (32.9%) MSM: 27/1,092 (2.5%)  HepA risk factors: 1,513/1,896 (79.8%) |
| Hu 2020 [70] | 2012–2018 | Review | NA (review) | NR for US population | NR | NR | NR | NR |
| Kaigh 2020 [37] | July 16 2019–October 8 2019 | Retrospective review | EHR data | NR | NR | NR | NR | NR |
| Kang 2020 [38] | November 1, 2016–October 31,2018 | Retrospective review | UCSDH, SDCPH | NR | NR | NR | NR | Among those receiving vaccinations (N=10,324): Homelessness: 1,385 (13%) Illicit drug use: 2,477 (24%) Alcohol abuse: 970 (9%) HepC infection: 104 (1%) HepB infection: 0 (0%) HIV infection: 585 (6%) Cirrhosis: 538 (5%) |
| Kronen 2022 [74] | October 2018–October 2019 | Quality improvement initiative | Hospital records | Preintervention, median (IQR): 58 (48, 65) years  Postintervention, median (IQR): 58 (48, 64) years | Preintervention, 116 (62%) Postintervention, 121 (62.4%) | Preintervention: White, 141 (75.4%) Black, 16 (8.6%) Hispanic, 10 (5.3%) Asian, 5 (2.7%)  Postintervention White, 140 (72.2%) Black, 13 (7.3%) Hispanic, 11 (5.7%) Asian, 6 (3.4%) | NR | NR |
| Kushel 2018 [75] | October 13 2017 (CA declaration of state of emergency)–NR | Outbreak response | NR | NR | NR | NR | NR | NR |
| Luder 2018 [72] | September 2014 through December 2015 | Pre-test/post-test study | Kroger pharmacies in Cincinnati/Dayton area | NR | NR | NR | NR | NR |
| McCosker 2022 [73] | NR | Systematic review | 23 published studies | NR | NR | NR | NR | NR |
| Montgomery 2022 [66] | June–August 2021 | Cross-sectional survey | Syringe service providers | NR | NR | NR | NR | NR |
| Montgomery 2021 [60] | NR | Editorial | NR | NR | NR | NR | NR | NR |
| NACCHO 2017 [61] | NR | Outbreak response webinar | NR | San Diego:  Median (range): 43 (5-96) years  Detroit:  Median: 42 years | San Diego: 363 (68%) Detroit: 297 (65%) | NR | San Diego County: HepC: 74/218 (17%) HepB: 22/427 (5.2%)  Detroit: HepC: 119 (27.5%) HepB: 13 (3.0%) | San Diego: Homeless and illicit drug use: 172 (32%) Homeless only: 87 (16%) Illicit drug use only: 64 (12%) Neither: 152 (28%) Unknown: 61 (11%)  Detroit: No substance abuse + No Homeless/Transient Living: 159 (36.8%) Homeless/Transient Living: 49 (11.3%) MSM: 21 (7.7%) Correctional Facility inmates: 27 (6.3%) Healthcare worker: 11 (2.5%) Food worker: 17 (3.9%) |
| Oren 2020 [64] | October2017– December 2017 | Grounded theory-based thematic analysis | Twitter | NA | NA | NA | NA | NA |
| Schwartz 2022 [54] | October 30, 2019– November 6, 2019 (8 days) | Descriptive study | Facebook | NA | NA | NA | NA | NA |
| Snyder 2019 [68] | January– October 2018 | Qualitative research study | Semi-structured interviews with health officials | NR | NR | NR | NR | NR |
| Sunshine 2019 [71] | 2016–2017 | Outbreak response | California Health Departments | NR | NR | NR | NR | NR |
| Talbott 2019 [77] | NR | Outbreak response | DPH | NR | NR | NR | NR | NR |
| Ungar 2019 [56] | Published: Feb 21, 2019 | News article | NR | NR | NR | NR | NR | NR |
| Viray 2019* [45] | June 11, 2016–October 22, 2016 | Outbreak response report | Medical records and other information (report of government investigation, findings, and public health response) | Median (range): 40 (16–80) years | 172 (59%) | Asian, 195 (69%) Native Hawaiian/Pacific Islander, 31 (11%) White, 29 (10%) Black, 1 (0%) Two or more races, 26 (9%) | NR | NR |
| Wooten 2019 [62] | March 8, 2017 (outbreak determined)–October 18, 2018 (outbreak declared over) | Outbreak response presentation | County of San Diego, Health & Human Services Agency, Public Health Services, Epidemiology & Immunization Services | NR | NR | NR | NR | NR |
| Wooten 2019 [63] | September 1, 2017 (declaration of local health emergency)–NR | Outbreak response report | NR | NR | NR | NR | NR | NR |
| Zellmer 2021 [78] | August 8, 2019 (MN DOH declare outbreak)–NR (April 18 2020 marks end of most of the state’s cases) | Outbreak response report | Statewide database | NR | NR | NR | NR | NR |

* Outbreak(s) occurred solely via food-borne transmission.

Abbreviations: BPA, best practices alert; CA, California; CDC, Centers for Disease Control and Prevention; DE, Delaware; DOH, Department of Health; DPH, Delaware Division of Public Health; ED, emergency department; EHR, electronic health record; HbsAg, hepatitis B surface antigen; HCV, hepatitis C virus; HepA, hepatitis A; HepB, hepatitis B; HepC, hepatitis C; HIV, human immunodeficiency virus; IQR, interquartile range; IV, intravenous; LGBTQ, lesbian, gay, bisexual, transgender, and queer; LMPHW, Louisville Metro Public Health and Wellness Department; MAVEN, Massachusetts Virtual Epidemiologic Network; MI, Michigan; MN, Minnesota; MSM, men who have sex with men; NA, not applicable; NR, not reported; NYC, New York City; PCR, polymerase chain reaction; SD, standard deviation; SDCPH, San Diego County Public Health; SUD, substance use disorder; TLR, targeted literature review; US, United States; UCSDH, University of California San Diego Health; UT, Utah; VA, Virginia.
